# Long‐distance dispersal is asymmetrical with respect to age, sex and breeding latitude in a long‐lived monogamous bird

**DOI:** 10.1111/1365-2656.70133

**Published:** 2025-09-14

**Authors:** E. H. J. (Lisenka) de Vries, Michiel P. Boom, Bart A. Nolet, Eelke Jongejans, Henk P. van der Jeugd

**Affiliations:** ^1^ Department of Animal Ecology Netherlands Institute of Ecology (NIOO‐KNAW) Wageningen The Netherlands; ^2^ Dutch Centre for Avian Migration and Demography, Netherlands Institute of Ecology (NIOO‐KNAW) Wageningen The Netherlands; ^3^ Department of Theoretical and Computational Ecology, Institute for Biodiversity and Ecosystem Dynamics University of Amsterdam Amsterdam The Netherlands; ^4^ Sovon Dutch Centre for Field Ornithology Nijmegen The Netherlands; ^5^ Department of Ecology, Radboud Institute for Biological and Environmental Sciences Radboud Univsersity Nijmegen The Netherlands

**Keywords:** adaptability, barnacle goose, Bayesian model, *Branta leucopsis*, flyway population, long‐distance dispersal, male‐biased natal dispersal, multistate joint live encounter–dead recovery model

## Abstract

1. Although relatively rare, long‐distance dispersal significantly impacts population persistence by facilitating range expansion, range shifts and genetic exchange. For individuals dispersing northwards, it may be a suitable adaptation strategy to escape negative effects of climate change on their original breeding sites.
2. In this study, we constructed a joint live encounter–dead recovery model under a Bayesian multistate framework to quantify long‐distance dispersal between the Barents Sea, Baltic Sea and North Sea subpopulations of the Russia/Germany and Netherlands flyway population of the barnacle goose (*Branta leucopsis*), using long‐term mark–recapture data of 22,413 individuals ringed between 1995 and 2023.
3. Long‐distance dispersal was strongly biased by age, sex and direction. Natal dispersal predominantly occurred in a northward direction, with 23.9% of juvenile males and 8.6% of juvenile females estimated to transition annually from the North Sea to the Barents Sea subpopulation. In contrast, breeding dispersal in the same direction in adults was minimal, estimated at only 0.49% and 0.21% for males and females respectively, and was not always distinguishable from temporary (moult‐) migrations.
4. Our model results were validated with data from 14 dispersing individuals, 9 of which were male, for whom the timing of breeding or moult was recorded. In all cases, dispersal was in a northward direction and the timing of breeding or moult of dispersers more closely resembled the timing of the receiving subpopulation than of the original subpopulation, but more so in males than in females.
5. Our results support the notion of strong male‐biased natal dispersal in monogamous waterbirds. Interestingly, despite substantial growth in the temperate breeding subpopulations during our study period, natal dispersal occurred predominantly in a northward direction at both individual and population levels. The unidirectional long‐distance dispersal observed is expected to result from the unique flyway structure, where subpopulations with large differences in population size mix during wintering. Additionally, we also highlight the adaptability of dispersers, showing that barnacle geese can adaptively switch migration on and off, and that plasticity in the timing of breeding and moult may be larger in males than in females. We argue that this could be an additional explanation for the predominantly male‐biased northward dispersal observed in barnacle geese.

## Graphical abstract

In this study, we constructed joint live encounter–dead recovery models under a Bayesian multistate framework to quantify long‐distance dispersal between barnacle goose subpopulations, using long‐term mark–recapture data. We demonstrate the existence of strong male‐biased unidirectional natal dispersal, resulting from low migratory connectivity and large differences in subpopulation sizes, and highlight the adaptability of dispersing individuals.

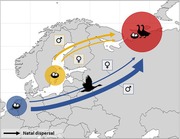

## 1 INTRODUCTION

Dispersal is a widespread demographic process that can be found across all taxa, affecting population dynamics, spatial distribution and the degree of genetic variation between populations (Greenwood, 1980). At the individual level, dispersal is often considered to be a strategy to improve breeding conditions by reducing competition for mates or resources and providing opportunities to find new sites or mates (Lagrange et al., 2014; Nevoux et al., 2013). As dispersal generally occurs more often in juveniles, a distinction is made between natal dispersal, that is, the permanent movement from the site or social group of birth to the site of first (potential) reproduction, and breeding dispersal, that is, the permanent movement between two successive breeding sites or groups (Clobert et al., 2001; Greenwood & Harvey, 1982). For natal dispersers, dispersal offers opportunities to settle in new habitats that are not yet saturated with experienced and dominant individuals, often leading to a higher fitness compared to philopatric individuals (Coulton et al., 2011). In addition, when habitat quality differs between sites, individuals can escape sites with poor conditions and increase individual fitness by dispersing towards sites with better conditions (Garant et al., 2007). If dispersal is effective, that is, the individual successfully reproduces after dispersing, the resulting gene flow aids in the avoidance of inbreeding (Clobert et al., 2001; Greenwood & Harvey, 1982). However, dispersal comes with costs to fitness, in addition to direct negative effects on survival experienced during dispersal, such as increased investment of energy and elevated risk of predation (Garant et al., 2007). Whereas philopatric individuals can benefit from knowledge on good nesting and foraging sites, dispersing individuals have to settle in unfamiliar environments (Coulton et al., 2011). Additionally, philopatric individuals may derive social benefits from the presence of kin, while those are lacking for unfamiliar (natal) dispersers (van der Jeugd, 2001). The outcome of the trade‐off between the costs and benefits ultimately determines the dispersal strategy and dispersal distance adopted by an individual. The infrequency of long‐distance dispersal events may imply that costs will often exceed the benefits, leading to the majority of dispersers settling in close proximity to their original site (Nevoux et al., 2013; van der Jeugd & Litvin, 2006).

Although relatively rare, the implications of long‐distance dispersal events at the population level may be profound. For instance, while gene flow reduces inbreeding, long‐distance dispersal is generally expected to lower genetic variation between populations by preventing local adaptation, directly affecting population persistence (Garant et al., 2007). Moreover, by facilitating range expansion, it enables the spread of (invasive) alien species to new areas (Bled et al., 2011; van den Bosch et al., 1992). However, it may also allow species to adequately respond to climate change (Rushing et al., 2015). Species that are incapable of adapting to their changing environment may mitigate negative effects by dispersing poleward to areas with better matching conditions. This adaptation‐through‐dispersal strategy could be particularly suitable for long‐distance migrants, which are especially susceptible to mismatches on their breeding grounds (Burger et al., 2013; Lamers et al., 2023).

Despite being rare, long‐distance dispersal remains notoriously difficult to study, especially in birds. Naturally, the high mobility of birds means they can settle virtually anywhere with suitable habitat (van Noordwijk, 1995), with the possibility of dispersal to areas far beyond the local study site. To correctly estimate dispersal, the probability of encountering a disperser should be constant across their entire dispersal range, including remote locations. However, as the probability of detecting a disperser declines with the dispersal distance (Koenig et al., 1996), low reporting rates are inevitable in some areas, and without correction, dispersal distance and the frequency of long‐distance dispersal will often be underestimated (Nathan et al., 2003). Large colony‐breeding birds like seabirds or geese may be more suitable for studying long‐distance dispersal using traditional marking methods, as their occurrence is strongly congregated in distinct colonies during the breeding season, making it relatively easy to monitor dispersal between breeding colonies.

In this study, we looked at long‐distance dispersal within the Russia/Germany & Netherlands flyway population of the barnacle goose (*Branta leucopsis*). Based on their breeding range, this population can be divided into three subpopulations, with long‐distance migrants breeding in Arctic Russia, short‐distance migrants breeding in the Baltic Sea area and sedentary birds breeding in the North Sea area (Madsen et al., 1999; Nagy et al., 2021). Individuals dispersing from one subpopulation to another have to adapt to both a new migration strategy and a different timing of breeding, as the temperate breeding birds breed significantly earlier than the Arctic breeding barnacle geese (van der Jeugd et al., 2009). The recent establishment of these subpopulations from one population breeding in Arctic Russia provides us with a ‘natural experiment’, enabling us to gain insight into the occurrence of long‐distance dispersal in barnacle geese. While incidental observations of colour‐ringed individuals indicate the existence of northwards dispersal between the three subpopulations (van der Jeugd, 2013; van der Jeugd & Litvin, 2006), this will be one of the first studies to quantify rates of long‐distance dispersal for this barnacle goose population in a Bayesian multistate model.

Our aim is therefore to quantify the extent of long‐distance dispersal between three subpopulations of barnacle geese within the same flyway, which vary in breeding latitude and migration behaviour. To do so, we constructed a multistate joint live encounter–dead recovery model based on a long‐term mark–recapture/resighting–recovery dataset. In addition to survival, multistate models allow the estimation of a ‘movement’ parameter, giving an unbiased estimate of the rate of transition from one state (here subpopulation) to another (Arnason, 1972, 1973; Barker et al., 2005). Furthermore, to look at the adaptability of dispersing individuals to differing timing of reproduction between subpopulations, we compared the timing of breeding or moult of a number of dispersed individuals to their original and receiving subpopulations.

## 2 MATERIALS AND METHODS

### 2.1 Study population

**FIGURE 1:**
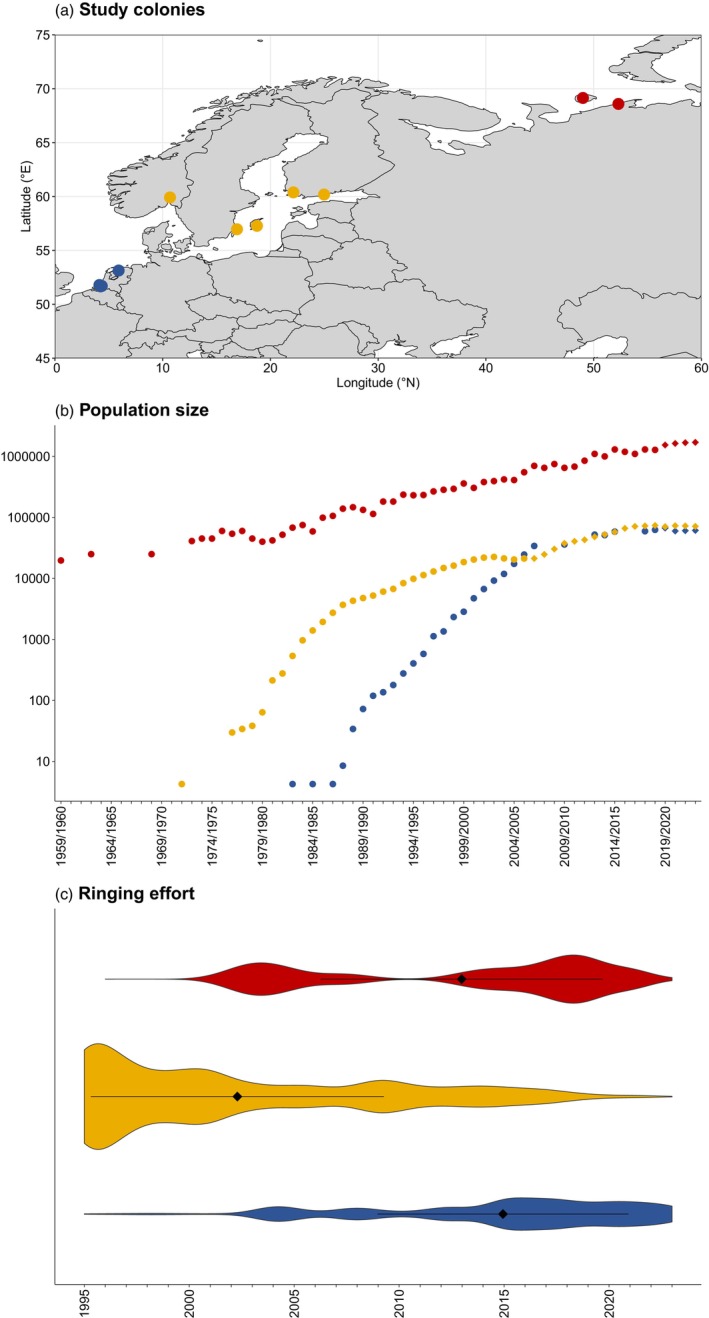
Locations of the main study colonies for the three subpopulations (a), development of the barnacle goose Russia/Germany and Netherlands flyway population and North Sea and Baltic Sea subpopulations (b) based on population counts, and an overview of the density in ringing effort in the three subpopulations (c). The Barents Sea subpopulation is represented here in red, the Baltic Sea subpopulation in yellow, while the North Sea subpopulation is represented in blue. Data on population sizes were collected from van der Jeugd et al. (2009), Koffijberg et al. (2020), Isaksen (2020), Nagy et al. (2021) and Jensen et al. (2022). Since flyway and subpopulation counts are unavailable after 2006 and incomplete for the Baltic Sea after 2002, these have been supplemented with sizes estimated in an integrated population model, indicated by diamond‐shaped dots (Baveco et al., 2020; Jensen et al., 2022).

The Russia/Germany and Netherlands flyway population of barnacle geese only numbered around 10,000 individuals in the early 1950s, but the introduction of a hunting ban in northwestern Europe and their ability to utilise intensively managed agricultural areas facilitated a strong increase in population numbers (Madsen et al., 1999). Currently, the population numbers around 1.4 million birds (Jensen et al., 2018, 2022). While the original population exclusively bred on islands off the coast of Arctic Russia, the breeding territories have been expanding to mainland Russia and former spring staging sites in the Baltic Sea region since the 1970s. Since the 1980s, the breeding population has further expanded to the original wintering sites in the Netherlands and neighbouring countries (Feige et al., 2008; Larsson et al., 1988; Madsen et al., 1999). As such, three subpopulations can now be distinguished based on their breeding areas: the migratory Barents Sea and Baltic Sea subpopulations and the resident North Sea subpopulations (Figure 1). The subpopulations still mix on the wintering sites in the North Sea region, although the distribution of the subpopulations across this area is not uniform (van der Jeugd et al., 2001).

### 2.2 Ringing and re‐encounter data

Barnacle geese have been the subject of long‐term studies throughout their range and have been consistently (colour‐)ringed during moult catches since the early 1980s as part of these (Figure 1a,c). The application of conspicuous colour marks aids in the identification of individuals without the need for recapturing (Marion & Shamis, 1977). Barnacle geese in the Baltic Sea region have been ringed on Gotland and Öland, Sweden, between 1984 and 2007 (Larsson et al., 1998; Larsson & Forslund, 1994; van der Jeugd & Larsson, 1998), in breeding colonies near Turku and Helsinki, Finland since the 1990s and in the small breeding colony near Oslo, Southeast Norway since 2002 (Kjell Isaksen, personal communication). In the Barents Sea region, barnacle geese have been ringed in study colonies on the Kolokolkova Bay salt marshes near the village of Tobseda since 2002 and on the island of Kolguev since 2006 (Eichhorn et al., 2006; Glazov et al., 2021; Mooij et al., 2011; van der Jeugd et al., 2003). Even with extensive ringing efforts, these study sites represent only a small proportion of the Barents Sea subpopulation (Lameris et al., 2023). In both the Barents Sea and Baltic Sea regions, encounters outside the study colonies are rare and mostly concern recoveries of dead individuals. In the North Sea area, barnacle geese have been ringed in small numbers during the breeding season since the 1980s. Since 2004, significant numbers have been ringed in the Dutch delta area (van der Jeugd, 2013), which comprised the main breeding colonies in the Netherlands. While monitoring of other Dutch breeding colonies is considerably less extensive, the widespread presence of volunteer observers throughout the Netherlands greatly increases the likelihood of resightings. Furthermore, small numbers of geese have been ringed in Belgium and Germany since 2015. In all regions, captured birds are sexed using cloacal inspection (Owen, 1980) and aged based on plumage as either juveniles or adults (birds 1 year old or older; Baker, 2016).

Since the 1950s, individuals have also been ringed while congregating on the wintering grounds in the North Sea area. The small number of individuals that were subsequently encountered in a breeding area in summer could be assigned to a subpopulation and were included in our analysis from that encounter onwards. As these birds are at least 1 year old when re‐encountered in summer, they only yield information on breeding dispersal, that is, the movement between two successive breeding sites (Clobert et al., 2001).

Ringing, recapture, resighting and dead recovery data were collected from the EURING Databank (du Feu et al., 2016) and the geese.org and Dutch Centre for Avian Migration and Demography (Vogeltrekstation) databases. Dead recovery data for Russia were supplemented with data collected directly from the Bird Ringing Centre Moscow (Konstantin Litvin, personal communication). Before inclusion in the analysis, the collected data were thoroughly checked (see Supporting Information for details). The cleaned dataset was constrained to data of individuals ringed during summer between 1995 and 2023. To further expand this dataset, individuals ringed in winter or before 1995 but encountered in subsequent summers after 1995 were included from that point onwards.

To be able to estimate the rate of dispersal between the different subpopulations of the barnacle geese, encounters of live individuals during summer were selected and assigned to one of the three geographical states (i.e. present during the breeding season in either the North Sea, Baltic Sea or Barents Sea subpopulation). For this, state‐specific ‘encounter windows’ were chosen, as the timing of live encounters of barnacle geese in summer strongly depends on the area (Table S1). Starting dates of the encounter windows for the North Sea and Baltic Sea subpopulations were chosen to minimise the probability that late migrants were wrongly included. For the small number of individuals that were seen in two states within an encounter window, the northernmost state was chosen. Meanwhile, dead recoveries are independent of states and occur continuously throughout the year. As such, dead recoveries of individuals in the interval between year *t* and year *t* + 1 were coded at year *t* + 1 (Duriez et al., 2009; Lebreton et al., 1999; LeTourneux et al., 2022).

### 2.3 Modelling framework

We used a joint live encounter–dead recovery model under a Bayesian multistate framework to estimate age‐ and sex‐specific dispersal rates between barnacle goose subpopulations using a multinomial likelihood formulation (Barker et al., 2005; Schaub & Kéry, 2021). This model is based on capture–mark–recapture/resighting‐recovery data for 22,413 barnacle geese ringed between 1995 and 2023 in the breeding areas described above.

Six nested models were fitted initially, with varying degrees of complexity (Table S2). Model selection was based on model fit and prior knowledge on survival and dispersal in barnacle geese (Baveco et al., 2020; van der Jeugd, 2013) and resulted in the choice for the most parsimonious model, which is described here. Model fit was assessed using goodness‐of‐fit tests using the program U‐Care version 3.3 (Choquet et al., 2009). As goodness‐of‐fit tests are currently unavailable for multistate models with a mixture of live encounters and dead recoveries, model fit was examined separately for live encounter and dead recovery data. Goodness of fit tests indicated the presence of (i) transience, which was dealt with by including age‐dependent survival, and (ii) (local) trap‐happiness, which was dealt with by including ring type and state in the recapture rate and by the inclusion of dead recovery data (see Supporting Information for details). To facilitate summarising the data into an M‐array formulation (Burnham et al., 1987), two age‐classes were included into the model as states. This resulted in a total of seven observable states and an unobservable absorbing ‘dead state’. The observable states are as follows: ‘Juvenile alive in North Sea area (1)’, ‘Juvenile alive in Baltic Sea area (2)’, ‘Juvenile alive in Barents Sea area (3)’, ‘Adult alive in North Sea area (4)’, ‘Adult alive in Baltic Sea area (5)’, ‘Adult alive in Barents Sea area (6)’ and ‘Recently dead (7)’, which specifies that the individual died in the interval from *t* − 1 to *t* and that it can no longer be recovered after *t* + 1 (Kéry & Schaub, 2011; Schaub & Kéry, 2021; Figures S1 and S2).

Within the model, transition rates were estimated as sex‐specific. As there is no evidence for differences in survival between sexes (Baveco et al., 2020; van der Jeugd, 2013), survival rates were estimated independent of sex. In this model, recapture rate is based on a combination of recaptures and resightings selected during a state‐specific encounter window. A group structure in the recapture rate based on ring types (only metal ring, coloured leg rings, coloured neckband or additional coloured mark added during recapture more than 1 year after the ringing event) was included to indirectly account for differences in the probability of resighting or recapturing an individual. That is, barnacle geese with metal rings are only recaptured, providing a recapture rate, while barnacle geese with coloured marks are generally (but not always, see Figure S3) resighted, providing a resighting rate. This same group structure was used for the estimation of the recovery rates. In addition, recapture rates were state‐specific. In summary, the following parameters were estimated in the model: the probability of survival from time *t* to *t* + 1 (conditional on age and state: S(state*age)), the probability of transition from a state at time *t* to another state at *t* + 1 (conditional on age and sex: Ψ(state*age*sex)), the probability of recapture at time *t* (conditional on state and ring type: p(state*ringtype)) and the probability that an individual died between time *t* − 1 and *t* and is found and reported (conditional on ring type: r(ringtype)). All parameters were set constant over time.

We fitted the multistate model using the program JAGS (Plummer, 2003) through the package jagsUI (Kellner, 2021) in R (R Core Team, 2023). We used vague uniformly distributed priors (U(0, 1)) for all parameters and ran three chains for 80,000 iterations, with a burn‐in of 40,000 at a thinning rate of 10. Convergence of the three MCMC chains for all monitored parameters was assessed using the Brooks–Gelman–Rubin $\hat{R}$ statistic (Brooks & Gelman, 1998) and by visually inspecting the trace plots.

In addition, the robustness of the parameter estimation in our model was tested using 13 simulation scenarios with varying survival, transition, recapture and recovery probabilities (see Supporting Information for details).

### 2.4 Dispersal in numbers of individuals

Transition estimates from a multistate model represent the yearly probability of an individual transitioning from one state to another. In order to obtain the actual number of individuals dispersing between the subpopulations, the transition estimates should be multiplied by the number of individuals in the different states. Mixing of the subpopulations on the wintering grounds in the North Sea area makes it impossible to count the subpopulations independently from each other during wintering, while counting during the breeding season when individuals are dispersed throughout each entire breeding range is not feasible. Therefore, posterior abundance estimates of subpopulation sizes in winter originating from subpopulation‐specific integrated population models, developed as part of the adaptive flyway management programme for this flyway population, were used (Baveco et al., 2020; Jensen et al., 2022). As there is no information on the sex ratio within the subpopulations, the abundance estimates were divided equally between the sexes (Table S7). All estimates were rounded up to represent whole numbers of individuals.

### 2.5 Timing of breeding and moult of dispersers

To gain insight into the phenological adaptability of barnacle geese, we investigated the timing of breeding or moult of individuals that dispersed between subpopulations. For this, we selected birds that were born, found with a nest or caught during moult in one subpopulation and were later recaptured during a moult catch or found with a nest in another subpopulation.

The onset of moult can be determined for individuals captured during moult catches, when flocks of flightless birds are rounded up in a corral and ringed. In addition to aging and sexing, the length of the ninth primary feather (counted in descending order) is measured using a ruler (±1 mm). Using this measurement, the onset of moult can be determined by assuming a primary growth of 7.00 mm/day for females and 7.44 mm/day for males, respectively (van der Jeugd et al., 2009). The day of onset of moult (rounded to integers) is then calculated by dividing the length of the primary feather by the growth rate and subtracting this number from the date of catching. The timing of breeding of the original and receiving subpopulations was based on hatch date. Only the lay date of the first egg was known for dispersing individuals with nests, not the fate of the nests. However, their hatch date could be estimated by adding 30 days to the lay date (van der Jeugd et al., 2009). We then compared the timing of breeding and moult of dispersing individuals relative to the mean timing of the original and the receiving subpopulation. For this, we used the mean and approximate 95% range (mean ± 2 SD) of timing in the population of origin and the annual mean and approximate 95% range (mean ± 2 SD) of the receiving population in the year in which the dispersing individual was encountered. The subpopulation‐specific mean on moult included the onset of moult of both breeding and non‐breeding birds. The timing of wing moult has been shown to vary between breeding and non‐breeding barnacle geese, particularly in the Arctic subpopulation (van der Jeugd et al., 2009). However, for all but one disperser encountered during moult, it is unclear whether it concerned breeding or non‐breeding individuals. Therefore, we opted to use the overall mean of the timing of moult for the three subpopulations. Data were tested for normality using a Shapiro–Wilk test, after which one‐sample *t*‐tests were performed to determine if the onset of moult of dispersers differed significantly from the timing of moult of the receiving subpopulations.

## 3 RESULTS

### 3.1 Robustness evaluation

Evaluation of model fit based on the Brooks–Gelman–Rubin statistic showed that $\hat{R}$ values were below 1.1 for all parameters in all simulation scenarios and were therefore deemed sufficiently well mixed. Overall, both the relative bias and root mean squared error (RMSE, Figures S5–S12), as well as the spread in both, were low across scenarios, indicating robustness. Particularly, Scenarios 2–4 showed consistently low bias and RMSE, underlining the model's ability to adequately estimate transition probabilities (Figures S5–S8). Although the relative bias and RMSE for the survival parameters was low for almost all scenarios, the negative relative bias and deviating RMSE observed for scenario 9 indicated an underestimation of survival when time‐varying survival was estimated in a model with constant survival (1.28%; Figures S9 and S10).

### 3.2 Sub‐population specific transition probabilities

**FIGURE 2:**
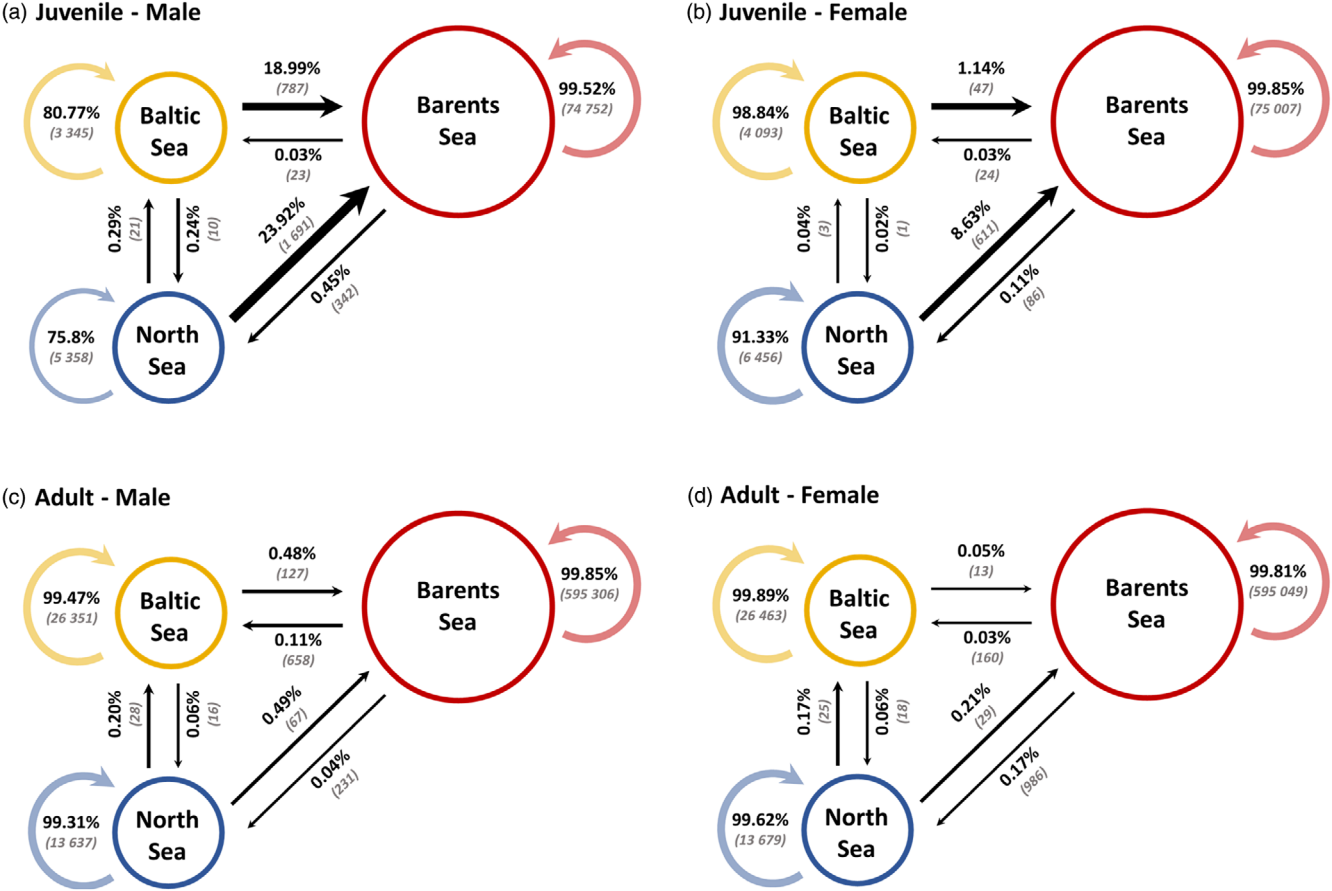
Age‐ and sex‐specific yearly transition probabilities between 1995 and 2023, given in percentages. Estimates have been rounded up to two decimals. Estimated age‐ and sex‐specific yearly numbers of dispersing individuals are given in the parentheses. These values were calculated using the estimated yearly transition rates and posterior abundance estimates for 2022 originating from a subpopulation‐specific integrated population model (see Table S7), and rounded up to represent whole numbers of individuals. Given are estimates for juvenile males (a), juvenile females (b), adult males (c) and adult females (d).

The results from our multistate model showed that long‐distance dispersal between subpopulations of the barnacle goose occurred between 1995 and 2023. However, transition rates differed substantially among subpopulations and between both sex and age classes (Figure 2; Table S8). Long‐distance dispersal was highest for juvenile males, with 23.9% (CRI 0.1515–0.3282) of all surviving juvenile males dispersing from the North Sea to the Barents Sea region and 19.0% (CRI 0.1066–0.2697) from the Baltic Sea to the Barents Sea region each year (Figure 2a). Although the estimated transition probability from the Barents Sea to the North Sea subpopulation was relatively low, due to the large differences in subpopulation size, on average 342 individuals would disperse towards the North Sea subpopulation each year. However, this is still considerably less than the 1691 individuals that are estimated to disperse in the opposite direction annually (Figure 2a). Long‐distance dispersal was substantially lower for juvenile females, with a yearly transition rate of 8.6% (CRI 0.0424–0.1411) from the North Sea to the Barents Sea region and of 1.1% (CRI 0–0.0328) from the Baltic Sea to the Barents Sea region in the past 28 years (Figure 2b). After accounting for subpopulation size differences, the number of dispersing juvenile females into the Barents Sea subpopulation remained lower than dispersal in the opposite direction. Each year, only 658 individuals disperse into the Barents Sea subpopulation (611 from the North Sea subpopulation and 47 from the Baltic Sea subpopulation), compared to 110 individuals dispersing southwards from the Barents Sea subpopulation each year (86 into the North Sea subpopulation and 24 into the Baltic Sea subpopulations; Figure 2b). For adults, yearly transition rates between subpopulations between 1995 and 2023 were considerably lower (Figure 2c,d) compared to juveniles. Even so, we still observed higher rates of yearly transition in the northwards direction, from the North Sea and Baltic Sea regions to the Barents Sea region, for adult males (Figure 2c) compared to adult females (Figure 2d). However, the yearly numbers of dispersing adult barnacle geese presented a contrasting picture. Due to the significantly larger size of the Barents Sea subpopulation, larger numbers of adults, both males and females, were estimated to disperse from the Barents Sea to the Baltic Sea (males: 658; females: 160) and North Sea (males: 231; females: 986) subpopulations each year than in the opposite direction (Figure 2c,d). However, the 95% Credible Interval for the estimated dispersal rates of individuals leaving the Barents Sea subpopulation reflects uncertainty, as the lower limit includes or approaches zero for both adult males and females.

As expected, yearly survival rates were generally lower in juveniles than in adults and differed between subpopulations (Figure S4A). For both ages, yearly survival was low in the North Sea subpopulation, estimated at 0.72 (± 0.006) and 0.53 (± 0.018) for adults and juveniles, respectively. An annual survival rate of 0.74 (± 0.0006) was estimated for adults belonging to the Barents Sea subpopulation, with an estimate of 0.53 (± 0.025) estimated for juveniles. Based on our model, individuals in the Baltic Sea subpopulation had the highest annual survival rate, 0.82 (± 0.002) and 0.58 (± 0.017) for adults and juveniles respectively. Unfortunately, we were unable to reliably estimate time‐dependent survival for the three subpopulations. As indicated by the results from our robustness checks, these time‐independent survival estimates are likely an underestimation.

### 3.3 Timing of breeding and moult of dispersers

We identified five females and nine males that dispersed to another subpopulation and were observed with a nest (3) and/or caught during moult (12) in their new subpopulation. All these ‘successful’ dispersal events took place in a northwards direction, from either the North Sea subpopulation to the Baltic Sea (3) or Barents Sea (3) subpopulations or from the Baltic Sea subpopulation to the Barents Sea subpopulation (8). For all dispersed individuals, the timing of breeding or moult matched more closely with the mean timing of the new subpopulation than that of the original subpopulation in the year in which they were observed (Table 1). In 14 cases (i.e. 93%), the timing of dispersed individuals was more similar to the mean of the receiving subpopulation than to the mean of the original subpopulation. Timing fell within the 95th percentile of the new subpopulation in all cases. In five cases (i.e. 33%), timing also fell close to the upper tail of the 95th percentile of the subpopulation of origin. Most individuals were caught as juveniles in their original subpopulation before dispersing (10, i.e. 67%), and were thereafter only observed with a nest or caught during moult in their new subpopulation, making within‐individual comparison of timing in the natal and receiving subpopulations impossible. Interestingly, dispersing males (*N* = 7) moulted, on average, in synchrony with the population mean of the receiving subpopulation, with a delay of 1.3 days (*t*_6_ = 0.563, *p* = 0.594). In contrast, dispersing females (*N* = 5) moulted significantly earlier, on average 6.4 days, than the population mean of their new subpopulation (*t*_4_ = −2.903, *p* = 0.044).

**TABLE 1:** Overview of the timing of hatching or moulting of dispersed individuals. Given is the subpopulation of origin (population where the individuals were born or caught first), and receiving subpopulation (where the bird was encountered after).

ID	Original population	Receiving population	Sex	Age at first capture	Year of encounter	Event	Timing (days of the year)	Timing original population (mean (95% range))	Timing receiving population (annual mean (95% range))	Mean difference original population	Mean difference receiving population
YCW2	BAL	BAR	M	J	2004	Hatch Date	195	150 (110–132)	**194 (182–199)**	45	**1**
L=Y1	NS	BAR	M	J	2008	Hatch Date	199	145 (101–134)	**197 (182–199)**	54	**2**
OTYY	NS	BAR	M	A	2015	Hatch Date	184	145 (101–134)	**184 (182–199)**	39	**0**
7003044	NS	BAL	M	J	1997	Onset of moult	190	**176 (152–191)**	**183 (174–195)**	14	**7**
7092460	NS	BAL	M	J	2000	Onset of moult	188	**176 (152–191)**	**184 (174–195)**	12	**4**
GJRP	NS	BAL	M	J	2000	Onset of moult	182	**176 (152–191)**	**184 (174–195)**	6	**‐2**
WFGP	BAL	BAR	F	J	2002	Onset of moult	200	185 (174–195)	**204 (191–217)**	15	**‐4**
YCW6	BAL	BAR	M	A	2003	Onset of moult	217	185 (174–195)	**207 (191–217)**	32	**10**
YHWH	BAL	BAR	F	A	2003	Onset of moult	197	185 (174–195)	**207 (191–217)**	12	**−10**
YCW2	BAL	BAR	M	J	2003	Onset of moult	201	185 (174–195)	**207 (191–217)**	16	**−6**
BPWH	BAL	BAR	M	J	2003	Onset of moult	208	185 (174–195)	**207 (191–217)**	23	**1**
L5OY	BAL	BAR	F	J	2005	Onset of moult	198	185 (174–195)	**199 (191–217)**	13	**−1**
OYW=	BAL	BAR	M	A	2005	Onset of moult	194	**185 (174–195)**	**199 (191–217)**	9	**−5**
GKLP	BAL	BAR	F	J	2008	Onset of moult	195	**185 (174–195)**	**208 (191–217)**	**10**	−13
OAY3	NS	BAR	F	J	2013	Onset of moult	204	176 (152–191)	**208 (191–217)**	28	**−4**

*Note*: Timing (in days of the year, 1 = Jan 1) is given for the dispersed individual in the new subpopulation. Information on the timing of the subpopulation of origin (mean and 95% range (mean ± 2 SD)) and receiving subpopulation (mean in the year the individual was observed and 95% range (mean ± 2 SD) of the subpopulation) is included to allow for a comparison of the timing of the dispersed individual to both subpopulations. Timing of the best corresponding individual is given in bold. Individual YCW2 was encountered on the nest in 2004 and during moulting in 2003 (with 2 fledged young), and is therefore included twice. Individual L = Y1 was also encountered on the nest in 2007, but is not included due to an unknown hatch date.

Abbreviations: BAL, Baltic Sea; BAR, Barents Sea; NS, North Sea.

## 4 DISCUSSION

The transition estimates of our joint live encounter–dead recovery model in a Bayesian multistate framework revealed the existence of extensive long‐distance dispersal, with a strong bias with respect to age, sex and breeding latitude. Natal dispersal of juvenile barnacle geese is asymmetrical and predominantly occurs in a northward direction. While almost a quarter of all surviving juvenile males were estimated to disperse from the resident North Sea subpopulation towards the migratory Arctic subpopulation, only around 8% of juvenile females dispersed in the same direction. Estimates for breeding dispersal in adults are much lower, but did show a similar asymmetric pattern, with predominantly male barnacle geese moving in a northward direction.

### 4.1 Male‐biased dispersal in barnacle geese

While dispersal tends to be predominantly female‐biased in most bird species, the opposite is observed for many waterfowl species, including swans, ducks and geese (Alison, 1975; Clarke et al., 1997; Coleman & Minton, 1979; Greenwood, 1980; Lessells, 1985; Lindberg et al., 1998). The existence of male‐biased dispersal in waterfowl, along with female‐biased philopatry, has often been linked to the division of roles between the sexes during the breeding season. While the role of a male passerine is often based on resource defence, which should aid in successfully attracting a mate or rearing offspring, in geese, the male's primary role involves mate defence against other males rather than resource defence. For the male, previous knowledge about the breeding area is therefore assumed to be less important (Clarke et al., 1997; Greenwood, 1980). In contrast, females are thought to benefit from familiarity with their breeding territory, enabling them to utilise information on high‐quality breeding areas as well as optimal foraging sites used to replenish body reserves during incubation breaks (Black et al., 2014; Clobert et al., 2001; Lessells, 1985; Prop et al., 1984). Female barnacle geese also exhibit a preference for nesting close to their mothers or sisters, seemingly benefitting from proximity to kin (Black et al., 2014; van der Jeugd et al., 2002). Evidence for a link between female philopatry in geese and breeding success is conflicted. While no positive effect was observed in black brant (*Branta bernicla nigricans*) (Lindberg & Sedinger, 1997), an increased future nesting success in relation to female philopatry and relatedness to neighbours was found for cackling geese (*Branta hutchinsii*) (Fowler, 2005). Still, it remains unclear whether philopatry improves breeding success or if breeding successfully encourages site fidelity.

Although male‐biased natal dispersal can thus be expected for barnacle geese, the observed northwards dispersal of especially juvenile males from the temperate to the Arctic breeding subpopulation can be explained by the unique structure of the flyway. The establishment of new breeding colonies in the former spring staging and wintering sites in the 1970s and 1980s resulted in a flyway with weak migratory connectivity, that is, three subpopulations with distinct breeding areas, but with a shared wintering location (Patel & Taylor, 2023). These subpopulations differ considerably in size, with the Barents Sea subpopulation being about 22–26 times larger than the other two subpopulations (Barents Sea subpopulation: ~1.6 million; Baltic Sea subpopulation: ~70,000; North Sea subpopulation: ~60,000; Figure 1b; Jensen et al., 2022). As pairs are often formed in winter and spring, when individuals from the different subpopulations intermingle (Owen et al., 1988; Rohwer & Anderson, 1988), the probability of an individual from one of the temperate breeding subpopulations encountering and subsequently pairing with an individual from the Barents Sea subpopulation should be much larger than the other way around (van der Jeugd, 2013). However, although evidence is limited, previous research has indicated that the three subpopulations are not distributed uniformly across the wintering area, with the wintering North Sea and Baltic Sea subpopulations remaining largely separated and with only limited mixing between the Barents Sea subpopulation and the two temperate breeding subpopulations (Ebbinge & van Biezen, 1987; van der Jeugd et al., 2001). Therefore, we calculated the predicted transition probabilities for juvenile males based on the yearly numbers of individuals given in Figure 2, under three scenarios of mixing: (1) fully random mixing between the three subpopulations in the entire wintering range, (2) random mixing between the Barents Sea subpopulation and the North Sea and Baltic Sea subpopulations in 10% of the wintering range and (3) random mixing between the Barents Sea subpopulation and the North Sea and Baltic Sea subpopulations in 1% of the wintering range. For the last two scenarios, there is no mixing between the two temperate breeding subpopulations (Table S11). The results show that the estimated transition probabilities are in line with scenarios of limited to very limited mixing: those from the North Sea towards the Barents Sea subpopulation lie somewhere between values calculated for the scenarios with random mixing in 1% and 10% of the wintering range, while the estimated transition probabilities from the Baltic Sea towards the Barents Sea subpopulation are in line with the calculated values based on a scenario with random mixing in 1% of the wintering range (Figure 2; Table S11). We therefore reject the hypothesis that natal dispersal rates are only a passive consequence of random mate choice among the set of encountered potential mates during wintering, and that other mechanisms must be at play that limit random mating at least to some extent. The gregarious nature of the barnacle goose means that their social connection is strong, with many social relationships spanning throughout and across years, both within and between sexes (Black et al., 2014; Kurvers et al., 2013, 2020). Most permanent pairings are formed after sexual maturity is reached in their second or third year, but many juveniles will enter in so‐called trial liaisons with several potential partners (van der Jeugd & Blaakmeer, 2001). In mixed flocks of Svalbard barnacle geese, Owen et al. (1988) found that males were (more than two times) more likely to pair with females from the same natal sites than expected when selecting mates randomly. Two forces that together shape the exact degree of mixing and pairing may thus lead to long‐distance natal dispersal in this species. It is likely that long‐distance dispersal resulting from mate choice during periods of population mixing might be a more general phenomenon in migrating animals. For instance, migratory waders are also known to exhibit low migratory connectivity, using distinct breeding sites, but mixing on wintering and stopover sites (e.g. piping plover *Charadrius melodus* (Ellis et al., 2021); dunlin *Calidris alpina* (Lopes et al., 2006); purple sandpiper *Calidris maritima* (LeBlanc et al., 2017)).

The large differences in subpopulation sizes also mean that the estimated transition probabilities do not accurately reflect the absolute number of dispersing individuals, specifically for adults. Due to the much larger Barents Sea subpopulations size, considerable numbers of adult barnacle geese, both male and female, appear to disperse from the Arctic to the temperate breeding subpopulations, even though the estimated transition probabilities show higher per individual probabilities of dispersing northwards than southwards. However, while specific care was taken to exclude late migrants (rather than dispersers) from the analysis, the possibility remains that this southwards movement of adult barnacle geese is directly linked to temporary moult migration of temperate breeding adult birds moulting in the Arctic and returning to their temperate breeding colonies in subsequent summers. Waterfowl undergo a complete wing moult, leaving them flightless and thus vulnerable for several weeks (Kjellén, 1994). Moult migration is thought to be undertaken by non‐ or failed‐breeders to escape towards areas deemed relatively safe and with better foraging opportunities, especially later in the breeding season (Glahder et al., 2007; Hupp et al., 2008; Kjellén, 1994; Salomonsen, 1968). Although it has not been formally documented for barnacle geese (but see Table S10 for anecdotal evidence based on encounters of 3 (colour‐)ringed individuals), long‐distance moult migrations have been observed in multiple other goose species (Aarvak & Øien, 2003; Glahder et al., 2007; Hupp et al., 2008; Kölzsch et al., 2019; Madsen & Mortensen, 1987; Piironen et al., 2021; Salomonsen, 1968). Distances can range up to several hundred kilometres (Kjellén, 1994), while greater white‐fronted geese breeding in the Barents Sea region were found to migrate as far as 2000 km to the Taimyr peninsula for moulting (Kölzsch et al., 2019).

Long‐term mark–recapture datasets like the ones we used here can provide valuable insight into processes taking place in large spatial and temporal space, but often come with limitations. The large differences in ringing effort between the subpopulations during the 28 years used in our model (Figure 1c) prevented us from calculating time‐dependent transition probabilities. It is to be expected that the magnitude of long‐distance dispersal may have changed as a result of the strong increase in subpopulation numbers during this time. It is also likely that, prior to the start of our dataset, the dominant direction of dispersal was southwards from the Barents Sea towards the Baltic and North Sea subpopulations, particularly during the early stages of establishment in the early 1970s and 1980s. Due to the absence of a consistent ringing effort in the three subpopulations during that time, it was not possible to investigate dispersal rates at the time of establishment of the temperate subpopulations using multistate models. However, Larsson et al. (1988) have demonstrated that the rapid increase in the number of breeding pairs observed on Gotland during the late 1970s and early 1980s could only be reached if immigration from the Barents Sea subpopulation occurred. While our results do not provide information on possible temporal changes in dispersal, they do give meaningful insight into directional differences in dispersal in this flyway population. For mark–recapture datasets that include colour‐marked individuals, the possible negative bias on the estimation of survival because of marker loss should be acknowledged. For long‐lived species like barnacle geese, marker loss has potentially large effects on the estimation of survival (Allen et al., 2019; Nelson et al., 1980). However, loss of coloured leg rings in barnacle geese generally appears to be low, with previous research showing an average loss of 0.35% annually between 1973 and 1986 (Rees et al., 1990). Moreover, rings are generally replaced upon recapture of birds with strongly worn or missing leg rings. While estimates of neck band loss in barnacle geese are lacking, an annual loss of 3.8% between 1997 and 2015 was found for greylag geese, resulting in an average underestimation of annual survival by 0.0342 (Schreven & Voslamber, 2022). As neckbands have only been used in small numbers (762 individuals) with the barnacle geese in our dataset since 2018, we do not expect there to be an effect on our estimation of annual adult or juvenile survival. Unfortunately, the direct impact of coloured leg rings on the survival of geese is currently unknown.

### 4.2 Adaptability of dispersers

Our results clearly point to a remarkable adaptability of the migratory syndrome in barnacle geese. Even decades after its establishment, birds born and raised as residents in the North Sea subpopulation are still able to (re‐)adopt a fully migratory lifestyle after dispersing to the Arctic. Migration patterns in geese are largely culturally inherited (Sutherland, 1998), enabling adaptations in migratory strategies to occur with relative ease. This is also evident in the rapid change in usage of spring‐staging sites in the Baltic region that has been observed in migratory barnacle geese (Eichhorn et al., 2009; Oudman et al., 2020; Tombre et al., 2019). However, although it is thus possible for former resident birds to adopt a migratory lifestyle, effective dispersal also requires a large adjustment in their timing of breeding. For dispersal to be effective and result in gene flow, an individual must be able to successfully reproduce after dispersing (Greenwood, 1980). Birds go through several stages throughout the year, and adjusting the timing of one stage in the annual cycle will generally have consequences for the timing of preceding or following stages (e.g. Tomotani, Muijres, et al., 2018; Tomotani, van der Jeugd, et al., 2018). Long‐distance migrants, like the barnacle goose, are less flexible in their timing as they go through more stages within a year (Wingfield, 2008). Since the establishment of the temperate breeding subpopulations in the 1970s and 1980s, the timing of reproductive stages between the temperate breeding and the Arctic breeding subpopulations has become increasingly divergent (van der Jeugd et al., 2009). Strong selection on early breeding in the temperate areas has led to the advancement of the breeding season, which means hatching of nests is now 6 weeks earlier than in the Arctic breeding subpopulation. Timing of wing moult has advanced as well, although to a lesser extent, and is now 2–4 weeks earlier in temperate breeding birds (van der Jeugd et al., 2009). The differences in timing of breeding and moult between the barnacle goose subpopulations may even negatively affect the fitness of dispersing individuals, as they may be unable to adequately adjust to the timing of the new subpopulation (Jonker et al., 2013). To determine the ability of dispersers to adjust their timing of breeding and moult, we identified nine males and five females that dispersed to another subpopulation, and for which information on timing of breeding and/or moult was available. Although sample sizes are small, this information still provides insight into the adaptability of barnacle geese to phenological conditions at different latitudes. Most dispersed birds were previously captured and marked as juveniles and are presumed to have dispersed at a young age. All 14 individuals were first captured in the North Sea or Baltic Sea region and subsequently recaptured in the Barents Sea region. Because the Arctic study area only represents a small fraction of the total breeding range of barnacle geese in the Russian Arctic (Lameris et al., 2023), it is remarkable that so many dispersed individuals were encountered here. In contrast, no disperser from the Arctic subpopulation was encountered in the temperate breeding subpopulations, even though the study areas contain a much larger proportion of those subpopulations. For both sexes, timing of dispersed individuals resembled the timing of the receiving subpopulation more closely in all but one case, indicating that both sexes are capable of adjusting the timing of annual cycle stages, although females showed a smaller shift in timing than males. This could indicate that females face greater constraints in shifting their timing of annual cycles, highlighting an additional factor that may contribute to the male‐biased long‐distance dispersal observed in barnacle geese. Additionally, the probability of dispersing successfully might be higher in the northward direction, as this requires a delay of the breeding stages rather than an advance. The physiological changes necessary to start the next stage (e.g. gonadal development, accumulation of body stores) are triggered by environmental cues, like photoperiod or temperature. While the onset of a breeding stage can be delayed by overriding environmental factors, individuals are limited in the advancement of breeding stages by the timing of the environmental cue and the time needed for the necessary physiological changes (Wingfield, 2008). Changes in heritable traits, as a result of selection, take place over multiple generations and are therefore generally slow. In comparison, phenotypically plastic traits can rapidly adjust to changing environmental conditions, even if not always ‘perfect’ (Charmantier & Gienapp, 2014). Lameris et al. (2018) concluded that although Arctic breeding barnacle geese are able to advance their arrival in response to earlier snowmelt, they are unable to sufficiently advance their lay dates to avoid a mismatch with the peak in food availability. As Arctic breeding barnacle geese are already unable to advance their timing of breeding adequately to optimise reproduction within their natal subpopulation, this raises the question of whether they would be able to keep pace with the selective pressures towards even earlier breeding in the temperate breeding subpopulations. Therefore, while southward dispersal may be probable, it is likely to be ineffective.

### 4.3 Implications of dispersal

While our multistate model shows considerable asymmetric natal dispersal of juvenile males in a northwards direction towards the Arctic breeding subpopulation, it remains uncertain whether this will impact the demography of the flyway population as a whole. The large size of the Arctic subpopulation means that even seemingly large immigration rates only represent a small proportion of its total recruitment, which may not significantly affect population growth rates (Lowe & Allendorf, 2010). Nevertheless, the amount of long‐distance dispersal we detected may still have genetic consequences. The identification of dispersed individuals with nests and even with fledged young shows that effective dispersal is possible. Even moderate exchange rates can affect genetic variation among populations, precluding speciation (Black et al., 2014). Long‐distance migrants like the barnacle goose are often argued to be especially susceptible to mismatches on their breeding grounds due to climate change, as they are unable to predict conditions while on their wintering grounds (Gill et al., 2014; Kölzsch et al., 2015). However, for migrants with low migratory connectivity, northwards long‐distance dispersal towards alternative breeding areas with more suitable conditions can enable species to escape the negative effects of the warming climate (Lameris et al., 2023), and such changes in migratory behaviour can be expected to progress more rapidly when transmitted culturally rather than genetically (Sutherland, 1998; van Noordwijk et al., 2006). For example, following a period of anthropogenic changes in their environment, pink‐footed geese *Anser brachyrhynchus* have developed a completely new migration route and established a distinct breeding population on the island of Novaya Zemlya, likely facilitated by the warming spring temperatures in the Arctic (Madsen et al., 2023). Thus, for barnacle geese, the gene flow into the Arctic subpopulation resulting from effective dispersal from the earlier breeding temperate subpopulations may subsequently accelerate the process of earlier breeding in response to earlier springs.

## AUTHOR CONTRIBUTIONS

Funding was obtained by B. A. Nolet and H. P. van der Jeugd. E. H. J. (Lisenka) de Vries, M. P. Boom and H. P. van der Jeugd conceived the ideas; and E. H. J. (Lisenka) de Vries and H. P. van der Jeugd designed the methodology; E. H. J. (Lisenka) de Vries, M. P. Boom and H. P. van der Jeugd collected the data; E. H. J. (Lisenka) de Vries analysed the data and led the writing of the manuscript. All authors aided in the interpretation of the data, contributed critically to drafts and gave final approval for publication.

## CONFLICT OF INTEREST STATEMENT

The authors have no conflicts of interest to declare.

## Supporting information


**Figure S1.** Transition probability matrix used to define the multistate joint live encounter–dead recovery model. Rows correspond to the departure state at time *t*, while columns represent the arrival state at *t* + 1. Subscripts represent age (juvenile vs. adult), departure and arrival state (A: North Sea subpopulation, B: Baltic Sea subpopulation, C: Barents Sea subpopulation).
**Figure S2.** Observation probability matrix used to define the multistate joint live encounter—dead recovery model. Rows correspond to the true state of the individual, while columns represent the observational states. Subscripts represent the arrival state (A: North Sea subpopulation, B: Baltic Sea subpopulation, C: Barents Sea subpopulation).
**Figure S3.** Number of individuals that, after ringing, were only recaptured (panel A), only resighted (panel B) or both recaptured and resighted (panel C). Numbers are given for each ring type and subpopulation.
**Figure S4.** Age‐ and subpopulation‐specific annual survival probabilities (panel A), ring type‐ and subpopulation‐specific recapture probabilities (panel B) and ring type‐specific recovery probabilities (panel C) between 1995 and 2023. In panel A, adult survival is represented by filled circles, while juvenile survival is represented by filled triangles. In panels B and C, coloured leg rings are represented by filled squares, metal rings by filled circles, neckbands are represented by a filled triangle, while both (additional coloured mark added during recapture) is represented by a filled diamond. Error bars signify 95% credible intervals.
**Figure S5.** Relative bias in estimates of transition rates between barnacle goose subpopulations for juvenile males and females estimated in 13 simulation scenarios. The spread of the deviation of the estimated parameter values relative to the input values of the simulations is represented in a boxplot, showing the median, 25th and 75th percentiles and any outlying points.
**Figure S6.** Root mean squared error (RMSE, $\sqrt{{\mathrm{sd}}^{2}+{\text{bias}}^{2}}$) for estimates of transition rates between barnacle goose subpopulations for juvenile males and females estimated in 13 simulation scenarios. The spread of the deviation of the estimated parameter values relative to the input values of the simulations is represented in a boxplot, showing the median, 25th and 75th percentiles and any outlying points.
**Figure S7.** Relative bias in estimates of transition rates between barnacle goose subpopulations for adult males and females estimated in 13 simulation scenarios. The spread of the deviation of the estimated parameter values relative to the input values of the simulations is represented in a boxplot, showing the median, 25th and 75th percentiles and any outlying points.
**Figure S8.**Root mean squared error (RMSE, $\sqrt{{\mathrm{sd}}^{2}+{\text{bias}}^{2}}$) for estimates of transition rates between barnacle goose subpopulations for adult males and females estimated in 13 simulation scenarios. The spread of the deviation of the estimated parameter values relative to the input values of the simulations is represented in a boxplot, showing the median, 25th and 75th percentiles and any outlying points.
**Figure S9.** Relative bias in estimates of age‐dependent survival rates for barnacle goose subpopulations estimated in 13 simulation scenarios. The spread of the deviation of the estimated parameter values relative to the input values of the simulations is represented in a boxplot, showing the median, 25th and 75th percentiles and any outlying points.
**Figure S10.** Root mean squared error (RMSE, $\sqrt{{\mathrm{sd}}^{2}+{\text{bias}}^{2}}$) for estimates of age‐dependent survival rates for barnacle goose subpopulations estimated in 13 simulation scenarios. The spread of the deviation of the estimated parameter values relative to the input values of the simulations is represented in a boxplot, showing the median, 25th and 75th percentiles and any outlying points.
**Figure S11.** Relative bias in estimates of re‐encounter (recaptures and resightings of live individuals) and recoveries for barnacle goose subpopulations, dependent on mark type, estimated in 13 simulation scenarios. The spread of the deviation of the estimated parameter values relative to the input values of the simulations is represented in a boxplot, showing the median, 25th and 75th percentiles and any outlying points.
**Figure S12.** Root mean squared error (RMSE, $\sqrt{{\mathrm{sd}}^{2}+{\text{bias}}^{2}}$) for estimates of re‐encounter (recaptures and resightings of live individuals) and recoveries for barnacle goose subpopulations, dependent on mark type, estimated in 13 simulation scenarios. The spread of the deviation of the estimated parameter values relative to the input values of the simulations is represented in a boxplot, showing the median, 25th and 75th percentiles and any outlying points.
**Table S1.** State‐specific ‘capture windows’ used to select encounters during summer. The capture windows are based on captures, recaptures and observations within the three subpopulations.
**Table S2.** Model structure of the initially fitted multistate models. Dependence of parameters is given between the parenthesis, with ‘.’ indicating a fully constant parameter. The used multistate model structure is given in bold.
**Table S3.** Results of the goodness‐of‐fit tests of the multistate live encounter data using the program U‐CARE.
**Table S4.** Results of test M.ITEC of the multistate live encounter data, including a group structure based on ring type in recaptures (coloured leg rings, metal ring, neckband or both (initially metal‐ringed, additional colour mark added at later recapture)) using the program U‐CARE .
**Table S5.** Results of the goodness‐of‐fit tests of the single state dead recovery data using the program U‐CARE.
**Table S6.** Overview of the constant used to simulate encounter histories based on 13 different scenarios. Simulated datasets were used to test parameter robustness of the constructed Bayesian multistate joint live encounter—dead recovery model. Subscripts signify the North Sea (A), Baltic Sea (B) and Barents Sea (C) subpopulations. For the first 10 scenarios, recapture (p) and recovery (r) probabilities were kept constant. ‘Both’ signifies individuals that were initially ringed with a metal ring, with an additional colour mark applied after at least a year. For the last 3 scenarios, recapture and recovery rates were either independent of ring type, state or both.
**Table S7.** Overview of the posterior abundance estimates in January in 2022 for adults and juveniles, as estimated from subpopulation specific IPMs, used to estimate absolute numbers of dispersing individuals. Also given is the 95% Credible Interval (Baveco et al., 2020; Jensen et al., 2022).
**Table S8.** Age‐ and sex‐specific yearly transition probabilities between 1995 and 2023 (posterior mean (credible interval)). Estimates have been rounded up to four decimals. Given are estimates for juvenile males (A), juvenile females (B), adult males (C) and adult females (D).
**Table S9.** Estimated age‐ and sex‐specific yearly numbers of dispersing individuals between 1995 and 2023, given in absolute numbers. Values were calculated using the estimated yearly transition rates and posterior abundance estimates for 2022 originating from a subpopulation‐specific Integrated population model (see Table S3). Given are estimated numbers for juvenile males (A), juvenile females (B), adult males (C) and adult females (D).
**Table S10.** Overview of encounters of 3 (colour‐)ringed barnacle geese giving anecdotal evidence of moult migration in adult barnacle geese. Given is the ring location and two subsequent locations of encounter during summer.
**Table S11.** Predicted transition probabilities for juvenile males, calculated using the total estimated numbers of individuals given in Figure 2. Transition probabilities were predicted based on three scenarios of mixing: 100% (fully random) mixing between the three subpopulations (A), 10% mixing between the Barents Sea subpopulation and the North Sea and Baltic Sea subpopulations (B), and 1% mixing between the Barents Sea subpopulation and the North Sea and Baltic Sea subpopulations (C). For the last two scenarios, there is no mixing between the two temperate breeding subpopulations. Percentages have been rounded up to two decimals.

## Data Availability

Data and source code available from the Dryad Digital Repository: https://doi.org/10.5061/dryad.8sf7m0d2p (de Vries et al., 2025).
