# Supplementary material for: Long‐distance dispersal is asymmetrical with respect to age, sex and breeding latitude in a long‐lived monogamous bird
Source: J Anim Ecol. 2025 Sep 14;94(11):2322–37. doi: 10.1111/1365-2656.70133 (PMC12586758; doi:10.1111/1365-2656.70133)

Transition

Adult − male

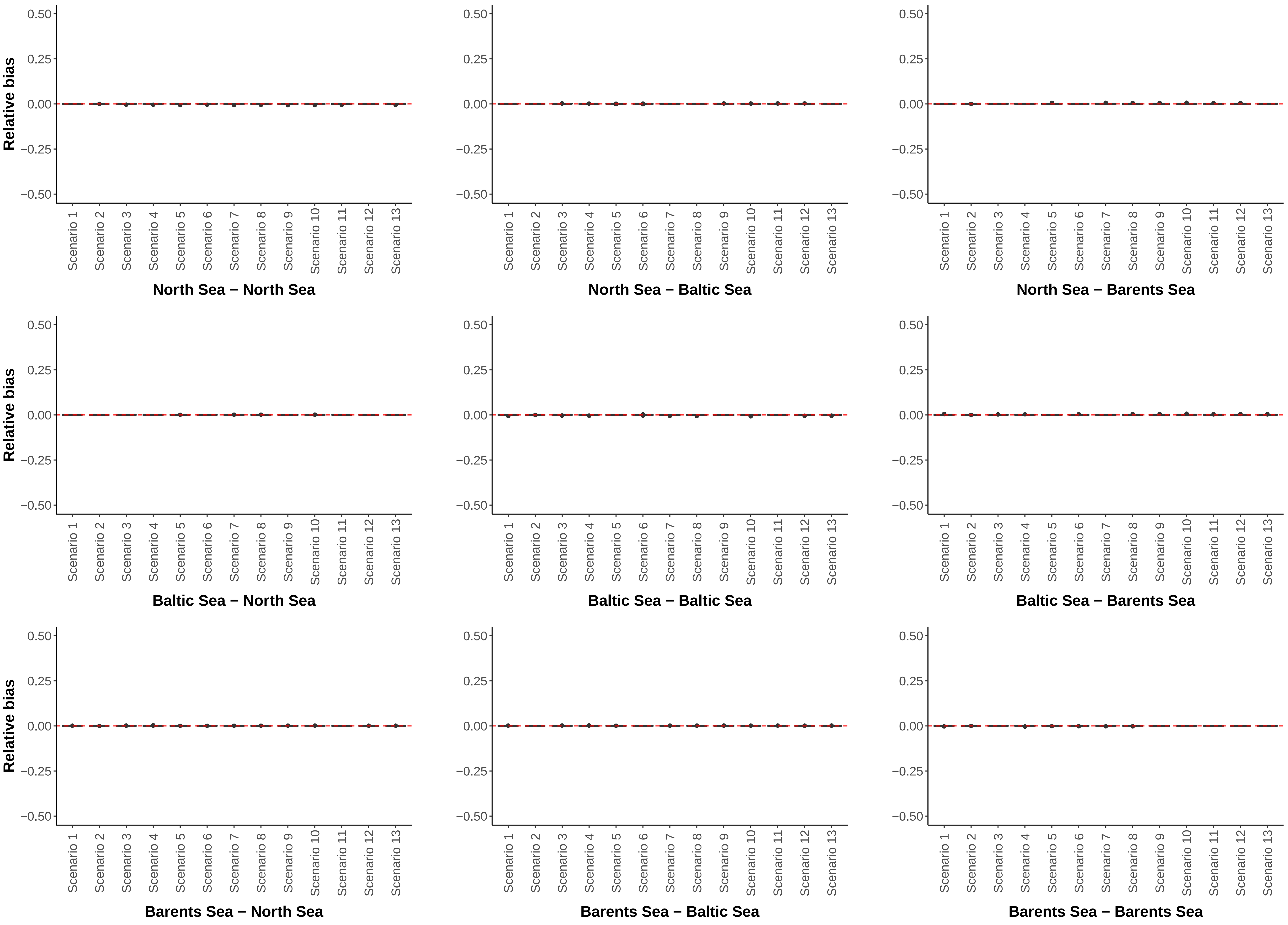

Adult − female

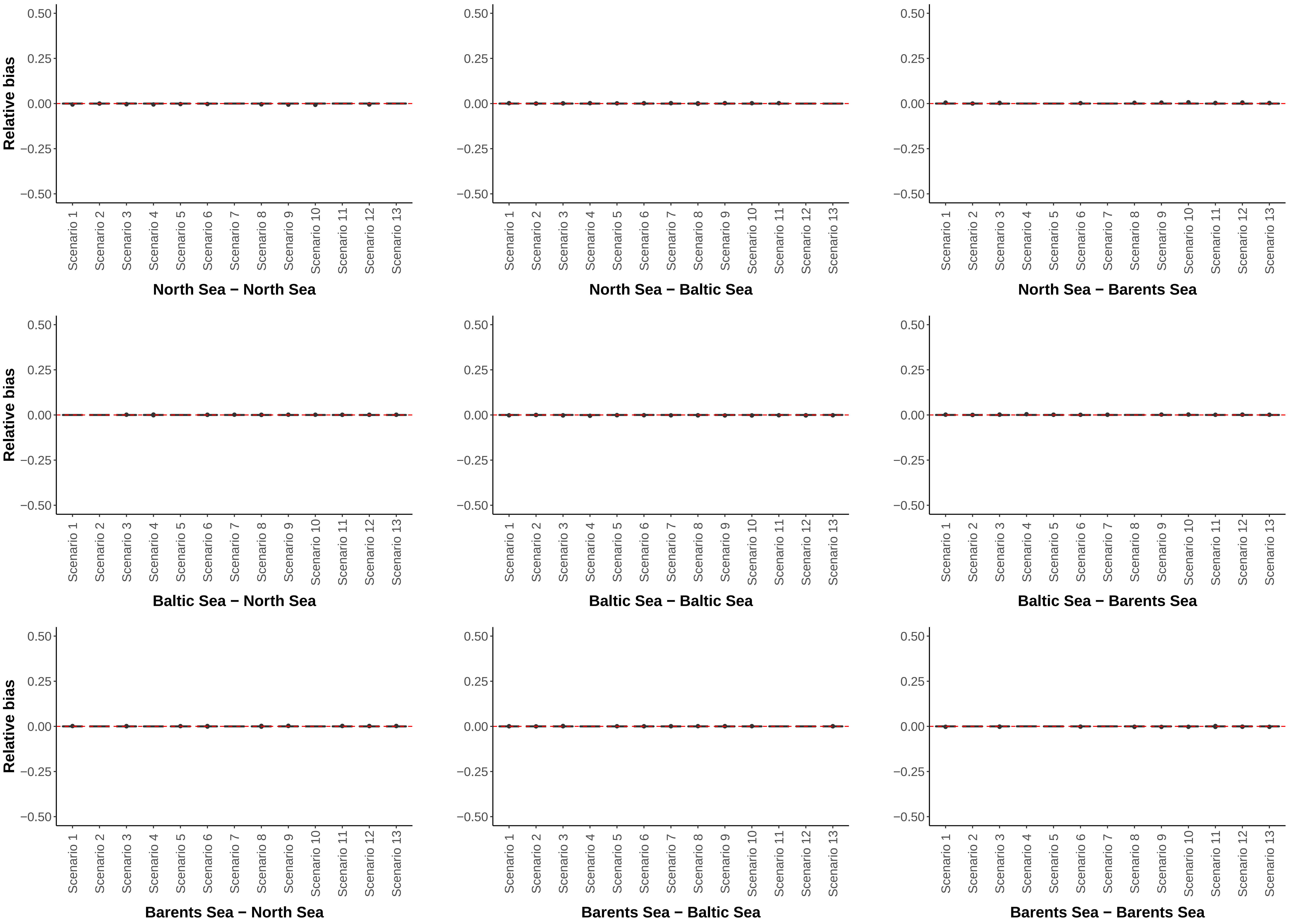

Supplement: Supplementary file 1 — Figure S1. Transition probability matrix used to define the multistate joint live encounter–dead recovery model. Rows correspond to the departure state at time t, while columns represent the arrival state at t + 1. Subscripts represent age (juvenile vs. adult), departure and arrival state (A: North Sea subpopulation, B: Baltic Sea subpopulation, C: Barents Sea subpopulation). Figure S2. Observation probability matrix used to define the multistate joint live encounter—dead recovery model. Rows correspond to the true state of the individual, while columns represent the observational states. Subscripts represent the arrival state (A: North Sea subpopulation, B: Baltic Sea subpopulation, C: Barents Sea subpopulation). Figure S3. Number of individuals that, after ringing, were only recaptured (panel A), only resighted (panel B) or both recaptured and resighted (panel C). Numbers are given for each ring type and subpopulation. Figure S4. Age‐ and subpopulation‐specific annual survival probabilities (panel A), ring type‐ and subpopulation‐specific recapture probabilities (panel B) and ring type‐specific recovery probabilities (panel C) between 1995 and 2023. In panel A, adult survival is represented by filled circles, while juvenile survival is represented by filled triangles. In panels B and C, coloured leg rings are represented by filled squares, metal rings by filled circles, neckbands are represented by a filled triangle, while both (additional coloured mark added during recapture) is represented by a filled diamond. Error bars signify 95% credible intervals. Figure S5. Relative bias in estimates of transition rates between barnacle goose subpopulations for juvenile males and females estimated in 13 simulation scenarios. The spread of the deviation of the estimated parameter values relative to the input values of the simulations is represented in a boxplot, showing the median, 25th and 75th percentiles and any outlying points. Figure S6. Root mean squared error (RMSE, [file JANE-94-2322-s001.zip › JANE_70133_f7_Figure_S7.pdf]
