# Supplementary material for: Long‐distance dispersal is asymmetrical with respect to age, sex and breeding latitude in a long‐lived monogamous bird
Source: J Anim Ecol. 2025 Sep 14;94(11):2322–37. doi: 10.1111/1365-2656.70133 (PMC12586758; doi:10.1111/1365-2656.70133)
Supplement: Supplementary file 1 — Figure S1. Transition probability matrix used to define the multistate joint live encounter–dead recovery model. Rows correspond to the departure state at time t, while columns represent the arrival state at t + 1. Subscripts represent age (juvenile vs. adult), departure and arrival state (A: North Sea subpopulation, B: Baltic Sea subpopulation, C: Barents Sea subpopulation). Figure S2. Observation probability matrix used to define the multistate joint live encounter—dead recovery model. Rows correspond to the true state of the individual, while columns represent the observational states. Subscripts represent the arrival state (A: North Sea subpopulation, B: Baltic Sea subpopulation, C: Barents Sea subpopulation). Figure S3. Number of individuals that, after ringing, were only recaptured (panel A), only resighted (panel B) or both recaptured and resighted (panel C). Numbers are given for each ring type and subpopulation. Figure S4. Age‐ and subpopulation‐specific annual survival probabilities (panel A), ring type‐ and subpopulation‐specific recapture probabilities (panel B) and ring type‐specific recovery probabilities (panel C) between 1995 and 2023. In panel A, adult survival is represented by filled circles, while juvenile survival is represented by filled triangles. In panels B and C, coloured leg rings are represented by filled squares, metal rings by filled circles, neckbands are represented by a filled triangle, while both (additional coloured mark added during recapture) is represented by a filled diamond. Error bars signify 95% credible intervals. Figure S5. Relative bias in estimates of transition rates between barnacle goose subpopulations for juvenile males and females estimated in 13 simulation scenarios. The spread of the deviation of the estimated parameter values relative to the input values of the simulations is represented in a boxplot, showing the median, 25th and 75th percentiles and any outlying points. Figure S6. Root mean squared error (RMSE, [file JANE-94-2322-s001.zip › jane70133-sup-0001-FigureS1-S12-TableS1-S11@supplements JAE-2025-00062.R2.docx]

**Supporting information for: Long-distance dispersal is asymmetrical with respect to age, sex and breeding latitude in a long-lived monogamous bird**

***Ringing and re-encounter data***

Observations occurring before the date of ringing were discarded. We also removed 21 individuals with encounters with an inaccuracy of date of more than three months on either side (EURING code ≥ 6; Euring, 2020). For encounters in Arctic Russia, an accuracy of date of more than 6 months on either side (EURING code ≥7, 28 individuals removed) was used, as barnacle geese can only be present there during summer. Live encounters after the dead recovery of an individual were scrutinized to determine the validity of the encounter and recovery. When more than 2 live encounters within three years after a dead recovery were registered, the recovery was determined to be invalid (21 recoveries). As it can be assumed that colour-ringed individuals are observed regularly, observations more than 6 years after the second last live encounter were also removed (881 observations).

***Goodness-of-fit tests***

Goodness-of-fit (GOF) tests were performed using the program U-CARE version 3.3 (Choquet et al., 2009). GOF tests are currently unavailable for multistate models with a mixture of live encounters and dead recoveries in U-CARE. We therefore examined GOF separately for live encounters and dead recoveries. The standard multistate GOF test in U-CARE was used for the live encounter data, as these are state-dependent. Since recoveries were independent of state, a single state GOF test was used for this dataset.

The multistate GOF test for live encounters revealed that both tests 3G, associated with transience, and tests M, associated with trap-dependence, were significant (Table S3). The existence of transience detected by test 3G.Sr, i.e., “new” individuals are re-encountered less than “old" individuals (Choquet et al., 2009), is commonly dealt with by including 2 age-classes in the survival parameter, as we did in our model. Unfortunately, sparseness of the data necessitated extensive pooling of data in test M.ITEC. Scrutinization of the pooled contingency tables revealed the possible existence of local trap-happiness, i.e., individuals encountered before have a higher re-encounter probability (Pradel et al., 2005). In our data set, live encounters consist of a combination of physical recaptures and observations of (colour-marked) individuals, both selected during a specific encounter window spanning the breeding season for each study region. The general ease of resighting a colour-ringed individual in comparison with the recapture of one, likely explains the found trap-happiness. When it is not possible to account for differences in recapture versus resighting probabilities directly, including a group structure in recapture rates based on ring types can be used as an indirect method. That is, barnacle geese with metal rings can only be recaptured, while barnacle geese with coloured leg rings or neckbands are generally (but not always, see Fig. S3) resighted. When including differences in ring type (4 groups: coloured leg rings, metal rings, coloured neckband and both (initially metal-ringed, additional colour mark added at later recapture)), test M.ITEC was significant for the “metal ring” and “coloured leg ring” groups, but not the “neckband” and “both” groups (Table S4). Examination of the pooled contingency tables indicates local trap-happiness is most obvious in individuals with coloured leg rings in the Barents Sea area and to a lesser degree also in the Baltic Sea area. The existence of local trap-happiness is not unexpected for either region, as the study sites in both areas represent only a small proportion of the respective subpopulations, particularly for the Barents Sea subpopulation. Live encounters outside of the study colonies are generally rare. Inclusion of dead recovery data should mitigate potential bias in survival estimates caused by the observed local trap-happiness (Frederiksen & Bregnballe, 2000; Frederiksen et al. 2014). Further capture heterogeneity caused by combining recapture and resighting data was accounted for by including both ring type and state in our estimation of the recapture rates in model.

For single state dead recovery data, only test 2.CTd, detecting an excess in immediate recoveries, and test 2.CLd, which detects an excess of recoveries in later years, can be interpreted (McCrea et al. 2014). Both tests were significant for our dead recovery data (Table S5), indicating the necessity of incorporation of 2 age-classes in survival (Choquet et al. 2020). Our multistate model is formulated as a multistate conditional Arnason-Schwarz (CAS) model (Arnason, 1972, 1973; Schwarz et al., 1993), in which it is not possible to account for a memory effect. The results from test WBWA in the multistate GOF test have therefore been disregarded. In addition, as the GOF tests were run separately for the two datasets, it was not possible to obtain an overall value of the overdispersion coefficient $\hat{c}$.

***Robustness evaluation***

Robustness of parameter estimation in our multistate model was tested using 13 simulation scenarios with varying survival and transition probabilities (Table S6). Scenarios 5 – 8 tested survival parameter estimation by simulating data using high and/or low survival estimates, derived from a previously developed joint live encounter – dead recovery model for barnacle geese (Baveco et al., 2020). Scenario 9 simulated data with time-varying survival by randomly generating values following a normal distribution using our estimated survival values as mean and a standard deviation of 0.5. Scenario 11 – 13 tested heterogeneity in recapture and recovery rates, by simulating data without differences in ring type and/or state.

For scenario 1 – 9, individual capture histories were simulated for 10 years with 100 individuals marked annually for 6 states: “ringed as juvenile in A”, “ringed as juvenile in B”, ringed as juvenile in C”, “ringed as adult in A”, ringed as adult in B”, “ringed as adult in C”, with sex-dependent transition rates. In scenario 10, individual capture histories were simulated for 10 years with the number of annually marked individuals randomly generated following a normal distribution with mean 100 and standard deviation 50. The generated values were rounded to whole absolute numbers. Recapture and recovery probabilities were kept constant for the first 10 scenarios, while survival and transition probabilities were kept constant for scenario 11 – 13.

The simulated data for each scenario were summed in a M-array formulation and fitted with the same model structure as used for the main analysis. Robustness of the parameters was evaluated by estimating the bias, i.e., the difference between the mean estimated value and inputted value, as well as the root mean square error (RMSE), i.e., $\sqrt{{sd}^{2}+{bias}^{2}}$ (Weegman et al., 2020). To get an indication of the spread of the deviation of the estimated values relative to the input values, the data simulation and analysis were run 100 times for each scenario.

***References***

Arnason, A. N. (1972). Parameter estimates from mark-recapture experiments on two populations subject to migration and death. *Researches on Population Ecology*, 13(2), 97–113.

<https://doi.org/10.1007/BF02521971>

Arnason, A. N. (1973). The estimation of population size, migration rates and survival in a stratified population. *Researches on Population Ecology*, 15(2), 1–8.

<https://doi.org/10.1007/BF02510705>

Baveco, J. M., Goedhart, P. W., Koffijberg, K., van der Jeugd, H. P., de Vries, L., Buij, R., & Nolet, B. A. (2020). *Development of an integrated population model for barnacle geese of the Russian management unit*. AEWA.

Choquet, R., Reboulet, A. M., Lebreton, J. D., Gimenez, O., & Pradel, R. (2020). U-CARE 3.3 User’s Manual (Version 3.3). CEFE.

Choquet, R., Rouan, L., & Pradel, R. (2009). Program E-Surge: a software application for fitting multievent models. In D. L. Thomson, E. G. Cooch, & M. J. Conroy (Eds.), Modeling demographic processes in marked populations (pp. 845–865). Springer US.

<https://doi.org/10.1007/978-0-387-78151-8_39>

EURING – The European Union for Bird Ringing. (2020). The EURING exchange code 2020. Helsinki, Finland.

Frederiksen, M., & Bregnballe, T. (2000). Evidence for density-dependent survival in adult cormorants from a combined analysis of recoveries and resightings. *Journal of Animal Ecology*, 69(5), 737–752. <https://doi.org/10.1046/j.1365-2656.2000.00435.x>

Frederiksen, M., Lebreton, J.-D., Pradel, R., Choquet, R., & Gimenez, O. (2014). REVIEW: Identifying links between vital rates and environment: a toolbox for the applied ecologist. *Journal of Applied Ecology*, 51(1), 71–81.

<https://doi.org/10.1111/1365-2664.12172>

Jensen, G. H., Johnson, F. A., Baveco, H., Koffijberg, K., Goedhart, P. W., McKenzie, R., & Madsen, J. (2022). *Population status and offtake assessment report 2022*. EGMP Technical Report No. 20.

McCrea, R. S., Morgan, B. J. T., & Pradel, R. (2014). Diagnostic goodness-of-fit tests for joint recapture and recovery models*. Journal of Agricultural, Biological, and Environmental Statistics*, 19(3), 338–356.

<https://doi.org/10.1007/s13253-014-0174-1>

Pradel, R., Gimenez, O., & Lebreton, J.-D. (2005). Principles and interest of GOF tests for multistate capture-recapture models. *Animal Biodiversity and Conservation*, 28(2), 189–204. <https://doi.org/10.32800/abc.2005.28.0189>

Schwarz, C. J., Schweigert, J. F., & Arnason, A. N. (1993). Estimating migration rates using tag-recovery data. *Biometrics*, 49(1), 177–193.

<https://doi.org/10.2307/2532612>

Weegman, M. D., Wilson, S., Alisauskas, R. T., & Kellett, D. K. (2020). Assessing bias in demographic estimates from joint live and dead encounter models. *PeerJ*, 8, e9382. https://doi.org/10.7717/peerj.9382

**Figures**

Figure S1: Transition probability matrix used to define the multistate joint live encounter – dead recovery model. Rows correspond to the departure state at time t, while columns represent the arrival state at t +1. Subscripts represent age (juvenile vs. adult), departure and arrival state (A: North Sea subpopulation, B: Baltic Sea subpopulation, C: Barents Sea subpopulation).

Figure S2: Observation probability matrix used to define the multistate joint live encounter – dead recovery model. Rows correspond to the true state of the individual, while columns represent the observational states. Subscripts represent the arrival state (A: North Sea subpopulation, B: Baltic Sea subpopulation, C: Barents Sea subpopulation).

Figure S3: Number of individuals that, after ringing, were only recaptured (panel A), only resighted (panel B) or both recaptured and resighted (panel C). Numbers are given for each ring type and subpopulation.

Figure S4: Age- and subpopulation-specific annual survival probabilities (panel A), ring type- and subpopulation-specific recapture probabilities (panel B) and ring type-specific recovery probabilities (panel C) between 1995 – 2023. In panel A, adult survival is represented by filled circles, while juvenile survival is represented by filled triangles. In panels B and C, coloured leg rings are represented by filled squares, metal rings by filled circles, neckbands are represented by a filled triangle, while both (additional coloured mark added during recapture) is represented by a filled diamond. Error bars signify 95% credible intervals.

Figure S5: Relative bias in estimates of transition rates between barnacle goose subpopulations for juvenile males and females estimated in 13 simulation scenarios. The spread of the deviation of the estimated parameter values relative to the input values of the simulations is represented in a boxplot, showing the median, 25^th^ and 75^th^ percentiles and any outlying points.

Figure S6: Root mean squared error (RMSE, $\sqrt{{sd}^{2}+{bias}^{2}}$) for estimates of transition rates between barnacle goose subpopulations for juvenile males and females estimated in 13 simulation scenarios. The spread of the deviation of the estimated parameter values relative to the input values of the simulations is represented in a boxplot, showing the median, 25th and 75th percentiles and any outlying points.

Figure S7: Relative bias in estimates of transition rates between barnacle goose subpopulations for adult males and females estimated in 13 simulation scenarios. The spread of the deviation of the estimated parameter values relative to the input values of the simulations is represented in a boxplot, showing the median, 25^th^ and 75^th^ percentiles and any outlying points.

Figure S8: Root mean squared error (RMSE, $\sqrt{{sd}^{2}+{bias}^{2}}$) for estimates of transition rates between barnacle goose subpopulations for adult males and females estimated in 13 simulation scenarios. The spread of the deviation of the estimated parameter values relative to the input values of the simulations is represented in a boxplot, showing the median, 25^th^ and 75^th^ percentiles and any outlying points.

Figure S9: Relative bias in estimates of age-dependent survival rates for barnacle goose subpopulations estimated in 13 simulation scenarios. The spread of the deviation of the estimated parameter values relative to the input values of the simulations is represented in a boxplot, showing the median, 25^th^ and 75^th^ percentiles and any outlying points.

Figure S10: Root mean squared error (RMSE, $\sqrt{{sd}^{2}+{bias}^{2}}$) for estimates of age-dependent survival rates for barnacle goose subpopulations estimated in 13 simulation scenarios. The spread of the deviation of the estimated parameter values relative to the input values of the simulations is represented in a boxplot, showing the median, 25^th^ and 75^th^ percentiles and any outlying points.

Figure S11: Relative bias in estimates of re-encounter (recaptures and resightings of live individuals)

and recoveries for barnacle goose subpopulations, dependent on mark type, estimated in 13 simulation scenarios. The spread of the deviation of the estimated parameter values relative to the input values of the simulations is represented in a boxplot, showing the median, 25^th^ and 75^th^ percentiles and any outlying points.

Figure S12: Root mean squared error (RMSE, $\sqrt{{sd}^{2}+{bias}^{2}}$) for estimates of re-encounter (recaptures and resightings of live individuals) and recoveries for barnacle goose subpopulations, dependent on mark type, estimated in 13 simulation scenarios. The spread of the deviation of the estimated parameter values relative to the input values of the simulations is represented in a boxplot, showing the median, 25^th^ and 75^th^ percentiles and any outlying points.

**Tables**

Table S1: State-specific “capture windows” used to select encounters during summer. The capture windows are based on captures, recaptures and observations within the three subpopulations.

| Subpopulation | Capture window |
| --- | --- |
| *North Sea* | June 6^th^ – August 14^th^ |
| *Baltic Sea* | June 1^th^ – September 4^th^ |
| *Barents Sea* | May 22^nd^ – August 18^th^ |

Table S2: model structure of the initially fitted multistate models. Dependence of parameters is given between the parenthesis, with “.” indicating a fully constant parameter. The used multistate model structure is given in bold.

| Model structure |
| --- |
| S(.)Psi(state)p(.)r(.) |
| S(state)Psi(state)p(state)r(.) |
| S(state*age)Psi(state*age)p(state)r(.) |
| S(state*age)Psi(state*age*sex)p(state)r(.) |
| S(state*age)Psi(state*age)p(state*ringtype)r(ringtype) |
| S(state*age)Psi(state*age*sex)p(state*ringtype)r(ringtype) |

Table S3: results of the goodness-of-fit tests of the multistate live encounter data using the program U-CARE.

| Test | χ^2^ | p-value | df | $\hat{\boldsymbol{c}}$ |
| --- | --- | --- | --- | --- |
| Overall | 6417.644 | 0.00 | 434 | 14.787 |
| WBWA | 18.878 | 0.004 | 6 | 3.14 |
| 3G.Sr | 4790.471 | 0.00 | 62 | 77.266 |
| 3G.Sm | 939.301 | 0.00 | 191 | 4.918 |
| M.ITEC | 462.554 | 0.00 | 65 | 7.116 |
| M.LTEC | 206.439 | 0.00 | 110 | 1.877 |

Table S4: results of test M.ITEC of the multistate live encounter data, including a group structure based on ring type in recaptures (coloured leg rings, metal ring, neckband or both (initially metal-ringed, additional colour mark added at later recapture) using the program U-CARE.

| Test | Ring type | χ^2^ | p-value | df | $\hat{\boldsymbol{c}}$ |
| --- | --- | --- | --- | --- | --- |
| M.ITEC | Coloured leg ring | 300.220 | 0.00 | 59 | 5.088 |
|  | Metal ring | 25.999 | 0.00 | 5 | 5.2 |
|  | Neckband | 0.001 | 0.976 | 1 | 0.001 |
|  | Both | 8.709 | 0.274 | 7 | 1.244 |

Table S5: results of the goodness-of-fit tests of the single state dead recovery data using the program U-CARE.

| Test | χ^2^ | p-value | df | $\hat{\boldsymbol{c}}$ |
| --- | --- | --- | --- | --- |
| 2.CTd | 116.1792 | 4.9305e-13 | 27 | 4.3 |
| 2.CLd | 149.0671 | 8.5927e-6 | 82 | 1.82 |

Table S6: Overview of the constant used to simulate encounter histories based on 13 different scenarios. Simulated datasets were used to test parameter robustness of the constructed Bayesian multistate joint live encounter – dead recovery model. Subscripts signify the North Sea (A), Baltic Sea (B) and Barents Sea (C) subpopulations. For the first 10 scenarios, recapture (p) and recovery (r) probabilities were kept constant. “Both” signifies individuals that were initially ringed with a metal ring, with an additional colour mark applied after at least a year. For the last 3 scenarios, recapture and recovery rates were either independent of ring type, state or both.

| Scenario | Survival | Transition (juvenile) | Transition  (adult) | Encounters |
| --- | --- | --- | --- | --- |
| 1  Estimates from multistate model | Juvenile  S_A_ = 0.534  S_B_ = 0.581  S_C_ = 0.531  Adult  S_A_ = 0.715  S_B_ = 0.817  S_C_ = 0.736 | Female  Ψ_AA_ = 0.9133  Ψ_AB_ = 0.0004  Ψ_AC_ = 0.0863  Ψ_BA_ = 0.0002  Ψ_BB_ = 0.9884  Ψ_BC_ = 0.0114  Ψ_CA_ = 0.001145  Ψ_CB_ = 0.000319  Ψ_CC_ = 0.998536  Male  Ψ_AA_ = 0.75798  Ψ_AB_ = 0.00287  Ψ_AC_ = 0.23915  Ψ_BA_ = 0.0024  Ψ_BB_ = 0.8077  Ψ_BC_ = 0.1899  Ψ_CA_ = 0.0045  Ψ_CB_ = 0.0003  Ψ_CC_ = 0.9952 | Female  Ψ_AA_ = 0.9962  Ψ_AB_ = 0.0017  Ψ_AC_ = 0.0021  Ψ_BA_ = 0.0006  Ψ_BB_ = 0.9989  Ψ_BC_ = 0.0005  Ψ_CA_ = 0.00165  Ψ_CB_ = 0.00027  Ψ_CC_ = 0.99808  Male  Ψ_AA_ = 0.9931  Ψ_AB_ = 0.002  Ψ_AC_ = 0.0049  Ψ_BA_ = 0.00058  Ψ_BB_ = 0.99465  Ψ_BC_ = 0.00477  Ψ_CA_ = 0.0004  Ψ_CB_ = 0.0011  Ψ_CC_ = 0.9985 | Coloured leg ring  p_A_ = 0.311  p_B_ = 0.607  p_C_ = 0.366  r = 0.06  Metal ring  p_A_ = 0.311  p_B_ = 0.657  p_C_ = 0.166  r = 0.059  Neckband  p_A_ = 0.515  p_B_ = 0.192  p_C_ = 0.001  r = 0.063  Both  p_A_ = 0.41  p_B_ = 0.321  p_C_ = 0.008  r = 0.048 |
| 2  No dispersal | Juvenile  S_A_ = 0.534  S_B_ = 0.581  S_C_ = 0.531  Adult  S_A_ = 0.715  S_B_ = 0.817  S_C_ = 0.736 | Female/Male  Ψ_AA_ = 1  Ψ_AB_ = 0  Ψ_AC_ = 0  Ψ_BA_ = 0  Ψ_BB_ = 1  Ψ_BC_ = 0  Ψ_CA_ = 0  Ψ_CB_ = 0  Ψ_CC_ = 1 | Female/Male  Ψ_AA_ = 1  Ψ_AB_ = 0  Ψ_AC_ = 0  Ψ_BA_ = 0  Ψ_BB_ = 1  Ψ_BC_ = 0  Ψ_CA_ = 0  Ψ_CB_ = 0  Ψ_CC_ = 1 | Coloured leg ring  p_A_ = 0.311  p_B_ = 0.607  p_C_ = 0.366  r = 0.06  Metal ring  p_A_ = 0.311  p_B_ = 0.657  p_C_ = 0.166  r = 0.059  Neckband  p_A_ = 0.515  p_B_ = 0.192  p_C_ = 0.001  r = 0.063  Both  p_A_ = 0.41  p_B_ = 0.321  p_C_ = 0.008  r = 0.048 |
| 3  High dispersal juveniles  Low dispersal adults | Juvenile  S_A_ = 0.534  S_B_ = 0.581  S_C_ = 0.531  Adult  S_A_ = 0.715  S_B_ = 0.817  S_C_ = 0.736 | Female  Ψ_AA_ = 0.9175  Ψ_AB_ = 0.041  Ψ_AC_ = 0.041  Ψ_BA_ = 0.041  Ψ_BB_ = 0.9175  Ψ_BC_ = 0.041  Ψ_CA_ = 0.041  Ψ_CB_ = 0.041  Ψ_CC_ = 0.9175  Male  Ψ_AA_ = 0.7462  Ψ_AB_ = 0.1269  Ψ_AC_ = 0.1269  Ψ_BA_ = 0.1269  Ψ_BB_ = 0.7462  Ψ_BC_ = 0.1269  Ψ_CA_ = 0.1269  Ψ_CB_ = 0.1269  Ψ_CC_ = 0.7462 | Female  Ψ_AA_ = 0.9992  Ψ_AB_ = 0.0004  Ψ_AC_ = 0.0004  Ψ_BA_ = 0.0004  Ψ_BB_ = 0.9992  Ψ_BC_ = 0.0004  Ψ_CA_ = 0.0004  Ψ_CB_ = 0.0004  Ψ_CC_ = 0.9992  Male  Ψ_AA_ = 0.9978  Ψ_AB_ = 0.0011  Ψ_AC_ = 0.0011  Ψ_BA_ = 0.0011  Ψ_BB_ = 0.9978  Ψ_BC_ = 0.0011  Ψ_CA_ = 0.0011  Ψ_CB_ = 0.0011  Ψ_CC_ = 0.9978 | Coloured leg ring  p_A_ = 0.311  p_B_ = 0.607  p_C_ = 0.366  r = 0.06  Metal ring  p_A_ = 0.311  p_B_ = 0.657  p_C_ = 0.166  r = 0.059  Neckband  p_A_ = 0.515  p_B_ = 0.192  p_C_ = 0.001  r = 0.063  Both  p_A_ = 0.41  p_B_ = 0.321  p_C_ = 0.008  r = 0.048 |
| 4  Low dispersal juveniles  High dispersal adults | Juvenile  S_A_ = 0.534  S_B_ = 0.581  S_C_ = 0.531  Adult  S_A_ = 0.715  S_B_ = 0.817  S_C_ = 0.736 | Female  Ψ_AA_ = 0.9988  Ψ_AB_ = 0.0006  Ψ_AC_ = 0.0006  Ψ_BA_ = 0.0006  Ψ_BB_ = 0.9988  Ψ_BC_ = 0.0006  Ψ_CA_ = 0.0006  Ψ_CB_ = 0.0006  Ψ_CC_ = 0.9988  Male  Ψ_AA_ = 0.9953  Ψ_AB_ = 0.00235  Ψ_AC_ = 0.00235  Ψ_BA_ = 0.00235  Ψ_BB_ = 0.9953  Ψ_BC_ = 0.00235  Ψ_CA_ = 0.00235  Ψ_CB_ = 0.00235  Ψ_CC_ = 0.9953 | Female  Ψ_AA_ = 0.9958  Ψ_AB_ = 0.0021  Ψ_AC_ = 0.0021  Ψ_BA_ = 0.0021  Ψ_BB_ = 0.9958  Ψ_BC_ = 0.0021  Ψ_CA_ = 0.0021  Ψ_CB_ = 0.0021  Ψ_CC_ = 0.9958  Male  Ψ_AA_ = 0.9958  Ψ_AB_ = 0.0021  Ψ_AC_ = 0.0021  Ψ_BA_ = 0.0021  Ψ_BB_ = 0.9958  Ψ_BC_ = 0.0021  Ψ_CA_ = 0.0021  Ψ_CB_ = 0.0021  Ψ_CC_ = 0.9958 | Coloured leg ring  p_A_ = 0.311  p_B_ = 0.607  p_C_ = 0.366  r = 0.06  Metal ring  p_A_ = 0.311  p_B_ = 0.657  p_C_ = 0.166  r = 0.059  Neckband  p_A_ = 0.515  p_B_ = 0.192  p_C_ = 0.001  r = 0.063  Both  p_A_ = 0.41  p_B_ = 0.321  p_C_ = 0.008  r = 0.048 |
| 5  High survival | Juvenile  S_A_ = 0.91  S_B_ = 0.91  S_C_ = 0.91  Adult  S_A_ = 0.97  S_B_ = 0.97  S_C_ = 0.97 | Female  Ψ_AA_ = 0.9133  Ψ_AB_ = 0.0004  Ψ_AC_ = 0.0863  Ψ_BA_ = 0.0002  Ψ_BB_ = 0.9884  Ψ_BC_ = 0.0114  Ψ_CA_ = 0.001145  Ψ_CB_ = 0.000319  Ψ_CC_ = 0.998536  Male  Ψ_AA_ = 0.75798  Ψ_AB_ = 0.00287  Ψ_AC_ = 0.23915  Ψ_BA_ = 0.0024  Ψ_BB_ = 0.8077  Ψ_BC_ = 0.1899  Ψ_CA_ = 0.0045  Ψ_CB_ = 0.0003  Ψ_CC_ = 0.9952 | Female  Ψ_AA_ = 0.9962  Ψ_AB_ = 0.0017  Ψ_AC_ = 0.0021  Ψ_BA_ = 0.0006  Ψ_BB_ = 0.9989  Ψ_BC_ = 0.0005  Ψ_CA_ = 0.00165  Ψ_CB_ = 0.00027  Ψ_CC_ = 0.99808  Male  Ψ_AA_ = 0.9931  Ψ_AB_ = 0.002  Ψ_AC_ = 0.0049  Ψ_BA_ = 0.00058  Ψ_BB_ = 0.99465  Ψ_BC_ = 0.00477  Ψ_CA_ = 0.0004  Ψ_CB_ = 0.0011  Ψ_CC_ = 0.9985 | Coloured leg ring  p_A_ = 0.311  p_B_ = 0.607  p_C_ = 0.366  r = 0.06  Metal ring  p_A_ = 0.311  p_B_ = 0.657  p_C_ = 0.166  r = 0.059  Neckband  p_A_ = 0.515  p_B_ = 0.192  p_C_ = 0.001  r = 0.063  Both  p_A_ = 0.41  p_B_ = 0.321  p_C_ = 0.008  r = 0.048 |
| 6  Low survival juvenile  High survival adults | Juvenile  S_A_ = 0.42  S_B_ = 0.42  S_C_ = 0.42  Adult  S_A_ = 0.97  S_B_ = 0.97  S_C_ = 0.97 | Female  Ψ_AA_ = 0.9133  Ψ_AB_ = 0.0004  Ψ_AC_ = 0.0863  Ψ_BA_ = 0.0002  Ψ_BB_ = 0.9884  Ψ_BC_ = 0.0114  Ψ_CA_ = 0.001145  Ψ_CB_ = 0.000319  Ψ_CC_ = 0.998536  Male  Ψ_AA_ = 0.75798  Ψ_AB_ = 0.00287  Ψ_AC_ = 0.23915  Ψ_BA_ = 0.0024  Ψ_BB_ = 0.8077  Ψ_BC_ = 0.1899  Ψ_CA_ = 0.0045  Ψ_CB_ = 0.0003  Ψ_CC_ = 0.9952 | Female  Ψ_AA_ = 0.9962  Ψ_AB_ = 0.0017  Ψ_AC_ = 0.0021  Ψ_BA_ = 0.0006  Ψ_BB_ = 0.9989  Ψ_BC_ = 0.0005  Ψ_CA_ = 0.00165  Ψ_CB_ = 0.00027  Ψ_CC_ = 0.99808  Male  Ψ_AA_ = 0.9931  Ψ_AB_ = 0.002  Ψ_AC_ = 0.0049  Ψ_BA_ = 0.00058  Ψ_BB_ = 0.99465  Ψ_BC_ = 0.00477  Ψ_CA_ = 0.0004  Ψ_CB_ = 0.0011  Ψ_CC_ = 0.9985 | Coloured leg ring  p_A_ = 0.311  p_B_ = 0.607  p_C_ = 0.366  r = 0.06  Metal ring  p_A_ = 0.311  p_B_ = 0.657  p_C_ = 0.166  r = 0.059  Neckband  p_A_ = 0.515  p_B_ = 0.192  p_C_ = 0.001  r = 0.063  Both  p_A_ = 0.41  p_B_ = 0.321  p_C_ = 0.008  r = 0.048 |
| 7  High survival juvenile  Low survival adult | Juvenile  S_A_ = 0.91  S_B_ = 0.91  S_C_ = 0.91  Adult  S_A_ = 0.79  S_B_ = 0.79  S_C_ = 0.79 | Female  Ψ_AA_ = 0.9133  Ψ_AB_ = 0.0004  Ψ_AC_ = 0.0863  Ψ_BA_ = 0.0002  Ψ_BB_ = 0.9884  Ψ_BC_ = 0.0114  Ψ_CA_ = 0.001145  Ψ_CB_ = 0.000319  Ψ_CC_ = 0.998536  Male  Ψ_AA_ = 0.75798  Ψ_AB_ = 0.00287  Ψ_AC_ = 0.23915  Ψ_BA_ = 0.0024  Ψ_BB_ = 0.8077  Ψ_BC_ = 0.1899  Ψ_CA_ = 0.0045  Ψ_CB_ = 0.0003  Ψ_CC_ = 0.9952 | Female  Ψ_AA_ = 0.9962  Ψ_AB_ = 0.0017  Ψ_AC_ = 0.0021  Ψ_BA_ = 0.0006  Ψ_BB_ = 0.9989  Ψ_BC_ = 0.0005  Ψ_CA_ = 0.00165  Ψ_CB_ = 0.00027  Ψ_CC_ = 0.99808  Male  Ψ_AA_ = 0.9931  Ψ_AB_ = 0.002  Ψ_AC_ = 0.0049  Ψ_BA_ = 0.00058  Ψ_BB_ = 0.99465  Ψ_BC_ = 0.00477  Ψ_CA_ = 0.0004  Ψ_CB_ = 0.0011  Ψ_CC_ = 0.9985 | Coloured leg ring  p_A_ = 0.311  p_B_ = 0.607  p_C_ = 0.366  r = 0.06  Metal ring  p_A_ = 0.311  p_B_ = 0.657  p_C_ = 0.166  r = 0.059  Neckband  p_A_ = 0.515  p_B_ = 0.192  p_C_ = 0.001  r = 0.063  Both  p_A_ = 0.41  p_B_ = 0.321  p_C_ = 0.008  r = 0.048 |
| 8  Low survival | Juvenile  S_A_ = 0.41  S_B_ = 0.41  S_C_ = 0.41  Adult  S_A_ = 0.79  S_B_ = 0.79  S_C_ = 0.79 | Female  Ψ_AA_ = 0.9133  Ψ_AB_ = 0.0004  Ψ_AC_ = 0.0863  Ψ_BA_ = 0.0002  Ψ_BB_ = 0.9884  Ψ_BC_ = 0.0114  Ψ_CA_ = 0.001145  Ψ_CB_ = 0.000319  Ψ_CC_ = 0.998536  Male  Ψ_AA_ = 0.75798  Ψ_AB_ = 0.00287  Ψ_AC_ = 0.23915  Ψ_BA_ = 0.0024  Ψ_BB_ = 0.8077  Ψ_BC_ = 0.1899  Ψ_CA_ = 0.0045  Ψ_CB_ = 0.0003  Ψ_CC_ = 0.9952 | Female  Ψ_AA_ = 0.9962  Ψ_AB_ = 0.0017  Ψ_AC_ = 0.0021  Ψ_BA_ = 0.0006  Ψ_BB_ = 0.9989  Ψ_BC_ = 0.0005  Ψ_CA_ = 0.00165  Ψ_CB_ = 0.00027  Ψ_CC_ = 0.99808  Male  Ψ_AA_ = 0.9931  Ψ_AB_ = 0.002  Ψ_AC_ = 0.0049  Ψ_BA_ = 0.00058  Ψ_BB_ = 0.99465  Ψ_BC_ = 0.00477  Ψ_CA_ = 0.0004  Ψ_CB_ = 0.0011  Ψ_CC_ = 0.9985 | Coloured leg ring  p_A_ = 0.311  p_B_ = 0.607  p_C_ = 0.366  r = 0.06  Metal ring  p_A_ = 0.311  p_B_ = 0.657  p_C_ = 0.166  r = 0.059  Neckband  p_A_ = 0.515  p_B_ = 0.192  p_C_ = 0.001  r = 0.063  Both  p_A_ = 0.41  p_B_ = 0.321  p_C_ = 0.008  r = 0.048 |
| 9  Temporal variation survival | Juvenile  S_A_ = rnorm(0.534, 0.5)  S_B_ = rnorm(0.581, 0.5)  S_C_ = rnorm (0.531, 0.5)  Adult  S_A_ = rnorm(0.715, 0.5)  S_B_ = rnorm(0.817, 0.5)  S_C_ = rnorm(0.736, 0.5) | Female  Ψ_AA_ = 0.9133  Ψ_AB_ = 0.0004  Ψ_AC_ = 0.0863  Ψ_BA_ = 0.0002  Ψ_BB_ = 0.9884  Ψ_BC_ = 0.0114  Ψ_CA_ = 0.001145  Ψ_CB_ = 0.000319  Ψ_CC_ = 0.998536  Male  Ψ_AA_ = 0.75798  Ψ_AB_ = 0.00287  Ψ_AC_ = 0.23915  Ψ_BA_ = 0.0024  Ψ_BB_ = 0.8077  Ψ_BC_ = 0.1899  Ψ_CA_ = 0.0045  Ψ_CB_ = 0.0003  Ψ_CC_ = 0.9952 | Female  Ψ_AA_ = 0.9962  Ψ_AB_ = 0.0017  Ψ_AC_ = 0.0021  Ψ_BA_ = 0.0006  Ψ_BB_ = 0.9989  Ψ_BC_ = 0.0005  Ψ_CA_ = 0.00165  Ψ_CB_ = 0.00027  Ψ_CC_ = 0.99808  Male  Ψ_AA_ = 0.9931  Ψ_AB_ = 0.002  Ψ_AC_ = 0.0049  Ψ_BA_ = 0.00058  Ψ_BB_ = 0.99465  Ψ_BC_ = 0.00477  Ψ_CA_ = 0.0004  Ψ_CB_ = 0.0011  Ψ_CC_ = 0.9985 | Coloured leg ring  p_A_ = 0.311  p_B_ = 0.607  p_C_ = 0.366  r = 0.06  Metal ring  p_A_ = 0.311  p_B_ = 0.657  p_C_ = 0.166  r = 0.059  Neckband  p_A_ = 0.515  p_B_ = 0.192  p_C_ = 0.001  r = 0.063  Both  p_A_ = 0.41  p_B_ = 0.321  p_C_ = 0.008  r = 0.048 |
| 10  Temporal variation in # individuals marked | Juvenile  S_A_ = 0.534  S_B_ = 0.581  S_C_ = 0.531  Adult  S_A_ = 0.715  S_B_ = 0.817  S_C_ = 0.736 | Female  Ψ_AA_ = 0.9133  Ψ_AB_ = 0.0004  Ψ_AC_ = 0.0863  Ψ_BA_ = 0.0002  Ψ_BB_ = 0.9884  Ψ_BC_ = 0.0114  Ψ_CA_ = 0.001145  Ψ_CB_ = 0.000319  Ψ_CC_ = 0.998536  Male  Ψ_AA_ = 0.75798  Ψ_AB_ = 0.00287  Ψ_AC_ = 0.23915  Ψ_BA_ = 0.0024  Ψ_BB_ = 0.8077  Ψ_BC_ = 0.1899  Ψ_CA_ = 0.0045  Ψ_CB_ = 0.0003  Ψ_CC_ = 0.9952 | Female  Ψ_AA_ = 0.9962  Ψ_AB_ = 0.0017  Ψ_AC_ = 0.0021  Ψ_BA_ = 0.0006  Ψ_BB_ = 0.9989  Ψ_BC_ = 0.0005  Ψ_CA_ = 0.00165  Ψ_CB_ = 0.00027  Ψ_CC_ = 0.99808  Male  Ψ_AA_ = 0.9931  Ψ_AB_ = 0.002  Ψ_AC_ = 0.0049  Ψ_BA_ = 0.00058  Ψ_BB_ = 0.99465  Ψ_BC_ = 0.00477  Ψ_CA_ = 0.0004  Ψ_CB_ = 0.0011  Ψ_CC_ = 0.9985 | Coloured leg ring  p_A_ = 0.311  p_B_ = 0.607  p_C_ = 0.366  r = 0.06  Metal ring  p_A_ = 0.311  p_B_ = 0.657  p_C_ = 0.166  r = 0.059  Neckband  p_A_ = 0.515  p_B_ = 0.192  p_C_ = 0.001  r = 0.063  Both  p_A_ = 0.41  p_B_ = 0.321  p_C_ = 0.008  r = 0.048 |
| 11  No difference in states between recaptures | Juvenile  S_A_ = 0.534  S_B_ = 0.581  S_C_ = 0.531  Adult  S_A_ = 0.715  S_B_ = 0.817  S_C_ = 0.736 | Female  Ψ_AA_ = 0.9133  Ψ_AB_ = 0.0004  Ψ_AC_ = 0.0863  Ψ_BA_ = 0.0002  Ψ_BB_ = 0.9884  Ψ_BC_ = 0.0114  Ψ_CA_ = 0.001145  Ψ_CB_ = 0.000319  Ψ_CC_ = 0.998536  Male  Ψ_AA_ = 0.75798  Ψ_AB_ = 0.00287  Ψ_AC_ = 0.23915  Ψ_BA_ = 0.0024  Ψ_BB_ = 0.8077  Ψ_BC_ = 0.1899  Ψ_CA_ = 0.0045  Ψ_CB_ = 0.0003  Ψ_CC_ = 0.9952 | Female  Ψ_AA_ = 0.9962  Ψ_AB_ = 0.0017  Ψ_AC_ = 0.0021  Ψ_BA_ = 0.0006  Ψ_BB_ = 0.9989  Ψ_BC_ = 0.0005  Ψ_CA_ = 0.00165  Ψ_CB_ = 0.00027  Ψ_CC_ = 0.99808  Male  Ψ_AA_ = 0.9931  Ψ_AB_ = 0.002  Ψ_AC_ = 0.0049  Ψ_BA_ = 0.00058  Ψ_BB_ = 0.99465  Ψ_BC_ = 0.00477  Ψ_CA_ = 0.0004  Ψ_CB_ = 0.0011  Ψ_CC_ = 0.9985 | Coloured leg ring  p_A_ = 0.428  p_B_ = 0.428  p_C_ = 0.428  r = 0.06  Metal ring  p_A_ = 0.378  p_B_ = 0.378  p_C_ = 0.378  r = 0.059  Neckband  p_A_ = 0.236  p_B_ = 0.236  p_C_ = 0.236  r = 0.063  Both  p_A_ = 0.2463  p_B_ = 0.2463  p_C_ = 0.2463  r = 0.048 |
| 12  No difference in ring type between encounters | Juvenile  S_A_ = 0.534  S_B_ = 0.581  S_C_ = 0.531  Adult  S_A_ = 0.715  S_B_ = 0.817  S_C_ = 0.736 | Female  Ψ_AA_ = 0.9133  Ψ_AB_ = 0.0004  Ψ_AC_ = 0.0863  Ψ_BA_ = 0.0002  Ψ_BB_ = 0.9884  Ψ_BC_ = 0.0114  Ψ_CA_ = 0.001145  Ψ_CB_ = 0.000319  Ψ_CC_ = 0.998536  Male  Ψ_AA_ = 0.75798  Ψ_AB_ = 0.00287  Ψ_AC_ = 0.23915  Ψ_BA_ = 0.0024  Ψ_BB_ = 0.8077  Ψ_BC_ = 0.1899  Ψ_CA_ = 0.0045  Ψ_CB_ = 0.0003  Ψ_CC_ = 0.9952 | Female  Ψ_AA_ = 0.9962  Ψ_AB_ = 0.0017  Ψ_AC_ = 0.0021  Ψ_BA_ = 0.0006  Ψ_BB_ = 0.9989  Ψ_BC_ = 0.0005  Ψ_CA_ = 0.00165  Ψ_CB_ = 0.00027  Ψ_CC_ = 0.99808  Male  Ψ_AA_ = 0.9931  Ψ_AB_ = 0.002  Ψ_AC_ = 0.0049  Ψ_BA_ = 0.00058  Ψ_BB_ = 0.99465  Ψ_BC_ = 0.00477  Ψ_CA_ = 0.0004  Ψ_CB_ = 0.0011  Ψ_CC_ = 0.9985 | Coloured leg ring  p_A_ = 0.38675  p_B_ = 0.44425  p_C_ = 0.13525  r = 0.0575  Metal ring  p_A_ = 0.38675  p_B_ = 0.44425  p_C_ = 0.13525  r = 0.0575  Neckband  p_A_ = 0.38675  p_B_ = 0.44425  p_C_ = 0.13525  r = 0.0575  Both  p_A_ =0.38675  p_B_ = 0.44425  p_C_ = 0.13525  r = 0.0575 |
| 13  No difference in ring type and state between encounters | Juvenile  S_A_ = 0.534  S_B_ = 0.581  S_C_ = 0.531  Adult  S_A_ = 0.715  S_B_ = 0.817  S_C_ = 0.736 | Female  Ψ_AA_ = 0.9133  Ψ_AB_ = 0.0004  Ψ_AC_ = 0.0863  Ψ_BA_ = 0.0002  Ψ_BB_ = 0.9884  Ψ_BC_ = 0.0114  Ψ_CA_ = 0.001145  Ψ_CB_ = 0.000319  Ψ_CC_ = 0.998536  Male  Ψ_AA_ = 0.75798  Ψ_AB_ = 0.00287  Ψ_AC_ = 0.23915  Ψ_BA_ = 0.0024  Ψ_BB_ = 0.8077  Ψ_BC_ = 0.1899  Ψ_CA_ = 0.0045  Ψ_CB_ = 0.0003  Ψ_CC_ = 0.9952 | Female  Ψ_AA_ = 0.9962  Ψ_AB_ = 0.0017  Ψ_AC_ = 0.0021  Ψ_BA_ = 0.0006  Ψ_BB_ = 0.9989  Ψ_BC_ = 0.0005  Ψ_CA_ = 0.00165  Ψ_CB_ = 0.00027  Ψ_CC_ = 0.99808  Male  Ψ_AA_ = 0.9931  Ψ_AB_ = 0.002  Ψ_AC_ = 0.0049  Ψ_BA_ = 0.00058  Ψ_BB_ = 0.99465  Ψ_BC_ = 0.00477  Ψ_CA_ = 0.0004  Ψ_CB_ = 0.0011  Ψ_CC_ = 0.9985 | Coloured leg ring  p_A_ = 0.322  p_B_ = 0.322  p_C_ = 0.322  r = 0.0575  Metal ring  p_A_ = 0.322  p_B_ = 0.322  p_C_ = 0.322  r = 0.0575  Neckband  p_A_ = 0.322  p_B_ = 0.322  p_C_ = 0.322  r = 0.0575  Both  p_A_ = 0.322  p_B_ = 0.322  p_C_ = 0.322  r = 0.0575 |

Table S7: Overview of the posterior abundance estimates in January in 2022 for adults and juveniles, as estimated from subpopulation specific IPMs, used to estimate absolute numbers of dispersing individuals. Also given is the 95% Credible Interval (Baveco et al., 2020; Jensen et al., 2022).

| Posterior Abundance estimates January | Adult | Juvenile |
| --- | --- | --- |
| *North Sea region* | 27 462.24622 (13 996.2 – 38 709.2) | 14 135.81455 (78.52 – 26 439.7) |
| *Baltic Sea region* | 52 984.77502 (34 633.8 – 66 035.5) | 8281.307922 (20.64 – 16 833.1) |
| *Barents Sea region* | 1 192 387.69 (1 060 740 – 1 313 590) | 150 232.4 (36 192.2 – 276 698) |

Table S8: Age- and sex-specific yearly transition probabilities between 1995 – 2023 (posterior mean (credible interval)). Estimates have been rounded up to four decimals. Given are estimates for juvenile males (A), juvenile females (B), adult males (C) and adult females (D).

| A.  Juvenile - male | To: |  |  |
| --- | --- | --- | --- |
| From: | *North Sea region* | *Baltic Sea region* | *Barents Sea region* |
| *North Sea region* | 0.7580 (0.6688 - 0.8456) | 0.0029 (0.0001 - 0.0106) | 0.2392 (0.1515 - 0.3282) |
| *Baltic Sea region* | 0.0024 (0.0001 - 0.0087) | 0.8077 (0.7278 - 0.8914) | 0.1899 (0.1066 - 0.2697) |
| *Barents Sea region* | 0.0045 (0.0003 - 0.0138) | 0.0003 (0.0000 - 0.0027) | 0.9952 (0.9858 - 0.9996) |

| B.  Juvenile -female | To: |  |  |
| --- | --- | --- | --- |
| From: | *North Sea region* | *Baltic Sea region* | *Barents Sea region* |
| *North Sea region* | 0.9133 (0.8586 - 0.9571) | 0.0004 (0.0000 - 0.0032) | 0.0863 (0.0424 - 0.1411) |
| *Baltic Sea region* | 0.0002 (0.0000 - 0.0019) | 0.9884 (0.9670 - 1.0000) | 0.0114 (0.0000 - 0.0328) |
| *Barents Sea region* | 0.0011 (0.0000 - 0.0088) | 0.0003 (0.0000 - 0.0027) | 0.9985 (0.9907 - 1.0000) |

| C.  Adult - male | To: |  |  |
| --- | --- | --- | --- |
| From: | *North Sea region* | *Baltic Sea region* | *Barents Sea region* |
| *North Sea region* | 0.9931 (0.9859 - 0.9977) | 0.0020 (0.0006 - 0.0044) | 0.0049 (0.0008 - 0.0119) |
| *Baltic Sea region* | 0.0006 (0.0002 - 0.0013) | 0.9947 (0.9913 - 0.9972) | 0.0048 (0.0022 - 0.0082) |
| *Barents Sea region* | 0.0004 (0.0000 - 0.0013) | 0.0011 (0.0003 - 0.0024) | 0.9985 (0.9970 - 0.9995) |

| D.  Adult - female | To: |  |  |
| --- | --- | --- | --- |
| From: | *North Sea region* | *Baltic Sea region* | *Barents Sea region* |
| *North Sea region* | 0.9962 (0.9929 - 0.9984) | 0.0017 (0.0005 - 0.0038) | 0.0021 (0.0004 - 0.0049) |
| *Baltic Sea region* | 0.0006 (0.0002 - 0.0013) | 0.9989 (0.9979 - 0.9995) | 0.0005 (0.0001 - 0.0013) |
| *Barents Sea region* | 0.0017 (0.0005 - 0.0034) | 0.0003 (0.0000 - 0.0009) | 0.9981 (0.9963 - 0.9994) |

Table S9: Estimated age- and sex-specific yearly numbers of dispersing individuals between 1995 – 2023, given in absolute numbers. Values were calculated using the estimated yearly transition rates and posterior abundance estimates for 2022 originating from a subpopulation-specific Integrated population model (see Table S3). Given are estimated numbers for juvenile males (A), juvenile females (B), adult males (C) and adult females (D).

| A.  Juvenile - male | To: |  |  |
| --- | --- | --- | --- |
| From: | *North Sea region* | *Baltic Sea region* | *Barents Sea region* |
| *North Sea region* | 5357.304 | 20.309 | 1690.294 |
| *Baltic Sea region* | 9.775 | 3344.423 | 786.456 |
| *Barents Sea region* | 341.686 | 22.550 | 74751.964 |

| B.  Juvenile - female | To: |  |  |
| --- | --- | --- | --- |
| From: | *North Sea region* | *Baltic Sea region* | *Barents Sea region* |
| *North Sea region* | 6455.319 | 2.554 | 610.034 |
| *Baltic Sea region* | 0.763 | 4092.792 | 47.100 |
| *Barents Sea region* | 85.991 | 23.977 | 75006.232 |

| C.  Adult - male | To: |  |  |
| --- | --- | --- | --- |
| From: | *North Sea region* | *Baltic Sea region* | *Barents Sea region* |
| *North Sea region* | 13636.970 | 27.329 | 66.821 |
| *Baltic Sea region* | 15.249 | 26350.727 | 126.414 |
| *Barents Sea region* | 230.962 | 657.215 | 595305.624 |

| D.  Adult - female | To: |  |  |
| --- | --- | --- | --- |
| From: | *North Sea region* | *Baltic Sea region* | *Barents Sea region* |
| *North Sea region* | 13678.766 | 24.023 | 28.331 |
| *Baltic Sea region* | 17.217 | 26462.990 | 12.183 |
| *Barents Sea region* | 985.882 | 159.603 | 595048.315 |

Table S10: Overview of encounters of 3 (colour-)ringed barnacle geese giving anecdotal evidence of moult migration in adult barnacle geese. Given is the ring location and two subsequent locations of encounter during summer.

| **Individual** | **Date** | **Location** | **Latitude, longitude** |
| --- | --- | --- | --- |
| *L1O7* | 13-08-2004 | Kolokolkova Bay, Russia | 68.58, 52.33 |
| *L1O7* | 19-07-2011 | Beltringharder Koog-Nord, Germany | 54.563, 8.872 |
| *L1O7* | 13-07-2015 | Kolokolkova Bay, Russia | 68.58, 52.33 |
| *OCY1* | 27-06-2012 | Westplaat Buitengronden, the Netherlands | 51.791, 4.127 |
| *OCY1* | 13-07-2015 | Tobseda, Russia | 68.857, 52.338 |
| *OCY1* | 02-07-2017 | Westplaat Buitengronden, the Netherlands | 51.789, 4.127 |
| *ML02778* | 08-08-2003 | Häme, Finland | 61.117, 25.55 |
| *ML02778* | 06-08-2004 | Kolokolkova Bay, Russia | 68.6, 52.467 |
| *ML02778* | 01-09-2006 | Kymi, Finland | 60.55, 27.167 |

Table S11: Predicted transition probabilities for juvenile males, calculated using the total estimated numbers of individuals given in Fig 2. Transition probabilities were predicted based on three scenarios of mixing: 100% (fully random) mixing between the three subpopulations (A), 10% mixing between the Barents Sea subpopulation and the North Sea and Baltic Sea subpopulations (B), and 1% mixing between the Barents Sea subpopulation and the North Sea and Baltic Sea subpopulations (C). For the last two scenarios, there is no mixing between the two temperate breeding subpopulations. Percentages have been rounded up to two decimals.

| **A: 100% mixing** | | | |
| --- | --- | --- | --- |
|  | **North Sea** | **Baltic Sea** | **Barents Sea** |
| **North Sea** | 8.19% | 4.80% | 87.02% |
| **Baltic Sea** | 8.19% | 4.80% | 87.02% |
| **Barents Sea** | 8.19% | 4.80% | 87.02% |

| **B: 10% mixing** | | | | | |
| --- | --- | --- | --- | --- | --- |
|  | **North Sea** | **Baltic Sea** | **Barents Sea (mixed with North Sea)** | **Barents Sea (mixed with Baltic Sea)** | **Barents Sea (mixed with Barents Sea)** |
| **North Sea** | 48.48% | - | 51.52% | - | - |
| **Baltic Sea** | - | 35.54% | - | 64.46% | - |
| **Barents Sea (mixed with North Sea)** | 48.48% | - | 51.52% | - | - |
| **Barents Sea (mixed with Baltic Sea)** | - | 35.54% | - | 64.46% | - |
| **Barents Sea (mixed with Barents Sea)** | - | - | - | - | 1 |

| **C: 1% mixing** | | | | | |
| --- | --- | --- | --- | --- | --- |
|  | **North Sea** | **Baltic Sea** | **Barents Sea (mixed with North Sea)** | **Barents Sea (mixed with Baltic Sea)** | **Barents Sea (mixed with Barents Sea)** |
| **North Sea** | 90.39% | - | 9.61% | - | - |
| **Baltic Sea** | - | 84.64% | - | 15.36% | - |
| **Barents Sea (mixed with North Sea)** | 90.39% | - | 9.61% | - | - |
| **Barents Sea (mixed with Baltic Sea)** | - | 84.64% | - | 15.36% | - |
| **Barents Sea (mixed with Barents Sea)** | - | - | - | - | 1 |
